# Integrating Global and National Knowledge to Select Medicines for Children: The Ghana National Drugs Programme

**DOI:** 10.1371/journal.pmed.1001449

**Published:** 2013-05-21

**Authors:** David Sinclair, Martha Gyansa-Lutterodt, Brian Asare, Augustina Koduah, Edith Andrews, Paul Garner

**Affiliations:** 1 Liverpool School of Tropical Medicine, Liverpool, United Kingdom; 2 Ghana National Drugs Programme, Accra, Ghana; 3 World Health Organization, Accra, Ghana

## Abstract

David Sinclair and colleagues discuss their experience at the Ghana National Drugs Programme reviewing the international evidence base for five priority pediatric medicines and report that applying the global recommendations to Ghana was not straightforward for any of the five medicines.

*Please see later in the article for the Editors' Summary*

**Summary Points**

- This paper reports the experience of the Ghana National Drugs Programme as they reviewed the international evidence base for five priority paediatric medicines.
- Applying the global recommendations to Ghana was not straightforward for any of the five medicines, regardless of the presence of high quality evidence of important clinical benefits.
- Four main factors generated debate and uncertainty in the committee: (1) effect unproven in African settings; (2) control group in trials not consistent with current practice; (3) little evidence on cost and cost effectiveness; and (4) limited supply chain.
- This project demonstrates why global recommendations should be presented alongside transparent descriptions of the evidence base, allowing policy groups to identify where, when, and how the interventions have been evaluated, and any factors limiting applicability.
- As many policy questions are relevant across sub-Saharan Africa, and policy makers are likely to encounter similar problems, we encourage regional collaboration on health technology assessment, and sharing of information and resources.

## Context

In 2011, the World Health Organization (WHO) Essential Medicines Programme published a list of “priority medicines” they considered essential for countries to achieve the Millennium Development Goals in child and maternal health [1]. This list was a subset of the model Essential Medicines List (EML), produced every 2 years by the WHO, which selects medicines on the basis of public health relevance, comparative effectiveness, safety, cost, and regulatory status [2].

The WHO model EML is adapted for use in Ghana by the Ghana National Drugs Programme of the Ministry of Health (MOH), and access to essential medicines is now largely financed through the National Health Insurance Scheme (NHIS). This scheme was established by the government of Ghana in 2003, and covers over 60% of the population [3]. Membership of the NHIS is through annual subscription, but free of charge to those under 18, over 70, pregnant, or the very poor, and members may access care through accredited public and private health care providers [4].

Following publication of the 2011 model EML it was noted that five of the priority paediatric medicines were not included in the 2010 Ghana EML: oral zinc sulphate for acute diarrhoea, injectable artesunate for severe malaria, topical chlorhexidine for preventing neonatal cord sepsis, dispersible oral amoxicillin for community acquired pneumonia, and oral and injectable caffeine citrate for neonatal apnoea [5].

Before adopting these medicines, the Ghana National Drugs Programme (GNDP) wanted to review the evidence base and how it applied to Ghana, using a transparent and evidence-informed approach, which further considered the local priorities, feasibility, and resource implications. In this paper we report on how the GNDP did this, and the difficulties experienced when interpreting and applying global recommendations to a national context.

## About This Project

The National Drugs Programme first prepared concise evidence summaries for each of the five WHO “priority” medicines. These five summaries were then used by the Ghana “Standard Treatment Guidelines” expert review committee in November 2011 (with representation from the NHIS and GNDP), to facilitate an open and informed discussion.

Training in the retrieval, appraisal, and interpretation of systematic reviews was provided for a selected team of Ministry of Health staff by specialists from the Liverpool School of Tropical Medicine. These staff then wrote the summaries, following a structure based on the work of the SUPPORT collaboration, summarising existing systematic reviews, rather than conducting new reviews [6]. The summaries addressed four main questions:

- What are the benefits and harms of [drug name]?
- What would be the public health impact of introducing [drug name] in Ghana?
- What are the resource implications to the country of introducing it?
- Is introduction currently feasible and acceptable in Ghana?

For evidence of benefits and harms participants searched the Cochrane Library and PubMed for existing systematic reviews. When more than one systematic review was found, the most reliable review was chosen on the basis of an evaluation of the search strategy and methods. When the most recent review was more than 2 years old an additional PubMed search was conducted for recently published randomized controlled trials. Confidence in the methods of systematic reviews was appraised using the AMSTAR checklist [7], and confidence in the results was appraised using the GRADE approach for assessing the quality of evidence [8]. For evidence on cost-effectiveness, participants reviewed the NHS Economics Database for economic evaluations relevant to each medicine, and appraised the methods using the CHEC-list [9]. The applicability of the systematic reviews and economic evaluations to Ghana was assessed following the guidance of the SUPPORT collaboration [10].

The potential public health impact of introducing each medicine was estimated by applying the relative mortality reductions from trial data to the best available national statistics for disease burden. A commentary on the costs and feasibility of introduction was prepared by reviewing international price guides for potential suppliers [11],[12], comparing the new drug price to the current alternative, and identifying any additional system or educational requirements for successful introduction [13].

A brief summary of the findings of each evidence summary is presented in Table 1. The full summaries are available as on-line supplements to this paper, and may provide useful templates for other countries.

**Table 1:** Summary of Ghana evidence summaries.

Priority Medicine (Formulation)	Benefits	Harms	Potential Public Health Impact	Feasibility	Resource Implications
**Zinc sulphate (Scored dispersible tablets 20 mg)**	Compared to placebo: • May shorten the duration of diarrhoea in children aged >6 months *(low quality evidence)*,• Probably has a larger effect in children with malnutrition *(moderate quality evidence)*.	Compared to placebo: • May increase the duration of diarrhoea In children aged <6 months *(low quality evidence)*,• Increases vomiting in both age groups *(high quality evidence)*.	An effect on child mortality has not been reliably demonstrated	Local manufacture is now in operation	Although a course of zinc sulphate is relatively cheap, the resource implications may be high due to the burden of disease.
**Artesunate (60 mg vial for injection plus 5% sodium bicarbonate buffer)**	Compared to quinine: • Lowers mortality *(high quality evidence)*	Compared to quinine: • Slightly increases neurological sequelae at hospital discharge *(high quality evidence)*,• Probably doesn't increase long-term neurological sequelae*(moderate quality evidence)*.	Potential to prevent up to 1,500 childhood deaths per year in Ghana	Dependent on identification of a reliable supplier	Change to artesunate may cost the Ghanaian National Malaria Programme an additional US$180,000 per year.
**Chlorhexidine (4% solution)**	Compared to dry cord care or soap and water: • May reduce neonatal mortality *(low quality evidence)*,• Probably reduces severe and moderate cord infections *(moderate quality evidence)*.	Compared to dry cord care or soap and water: • None known	Potential to prevent up to 4,500 neonatal deaths per year in Ghana	To achieve the effect seen in the trial mothers were visited at home six times, which may not be feasible in Ghana	No economic evaluations were found
**Amoxicillin (500 mg/250 mg scored dispersible tablets)**	Compared to suspension: • Improved dose accuracy,• Longer shelf-life with no need for refrigeration,• Less bulky for transport and less susceptible to high temperatures.	Compared to suspension: • None known	Probably minimal	Dependent on identification of a reliable supplier	Could represent a cost-saving dependant on supplier
**Caffeine citrate (Injection and oral solution 20 mg/ml; equivalent to 10 mg caffeine base/ml)**	Compared to placebo: • Probably reduces the risk of death >or major disability by late infancy *(moderate quality evidence)*,• Reduces the risk of chronic lung disease *(high quality evidence)*,• Probably reduces the risk of cognitive delay *(moderate quality evidence)*.	Compared to placebo/theophylline: • Caffeine probably has fewer side-effects than theophylline (*moderate quality evidence*)	Prematurity is considered the second major cause of infant mortality in Ghana, however reliable estimates of burden of neonatal apnoea were unavailable	There are currently no international suppliers of a suitable product	No price estimates or economic evaluations were found

## Our Experience

Applying global recommendations to Ghana was not straightforward for any of the five medicines, regardless of the presence of high quality evidence of important clinical benefits (Table 2). We have summarised the four key factors that generated debate and uncertainty in the committee, and tempered automatic adoption of the five medicines.

**Table 2:** Problems applying the global recommendations to Ghana.

Priority Medicine	Current WHO Recommendation [Source]	Problems Encountered in Applying the Global Evidence Base to Ghana	Panel Consideration and Decision
**Zinc sulphate** (Scored dispersible tablets 20 mg)	• For children aged <1 years with acute diarrhoea: 10 mg once daily for 14 days• For children age >1 years with acute diarrhoea: 20 mg once daily for 14 days [14].	• Most trial research is from Asian countries at high risk of zinc deficiency.• No effect has been seen in African trials from settings at moderate risk of zinc deficiency.• There is significant heterogeneity in the magnitude of the benefit of supplementation.	• The effect is largest and most consistent in children with signs of moderate malnutrition.• Dispersible zinc tablets were added to the EML on the basis of very high levels of malnutrition among children aged <5 years in northern and rural areas.
**Artesunate** (60 mg vial for injection plus 5% sodium bicarbonate buffer)	• For all children under 5 with severe malaria: 2.4 mg/kg on admission, at 12 hours, 24 hours, and then daily until oral therapy tolerated [19].	• The evidence of the superiority of artesunate over quinine is well documented but artemether is currently one of the most widely used injectable antimalarials in Ghana.• The WHO Malaria Treatment Guideline does not consider or offer advice on the cost or feasibility of introducing artesunate.• Ghanaian estimates of incidence and mortality of severe malaria appear unreliable, which in turn makes estimates of the potential public health impact and cost unreliable.	• Artesunate and artemether were compared indirectly by noting that artemether appears equivalent to quinine, and quinine is inferior to artesunate.• Artesunate was added to Ghana EML and for discussion with Ghana Malaria Control Programme for consideration as first line treatment in Ghana.• The change to artesunate will not be made until a reliable supply of a high quality, affordable product is assured.
**Chlorhexidine** (4% solution)	• For all neonates: Apply daily to umbilical stump [1].	• The only available evidence for chlorhexidine is from a single trial in Nepal.• This trial involved intensive home visits during the post-natal period which may not be feasible in Ghana.• The cord care received by the control groups in the single trial is different to current practice in Ghana.	• The panel considered the evidence inadequate to adopt chlorhexidine nationally.• Large-scale effectiveness studies are underway in Africa and chlorhexidine will be reviewed again once these results are available.
**Amoxicillin** (500 mg/250 mg scored dispersible tablets)	• For all children under 5 with community acquired pneumonia: 25 mg/kg twice daily for 3 to 5 days [20].	• While dispersible tablets offer clear logistical benefits over suspensions, the WHO recommendation also includes a change in frequency and duration of treatment, with little evidence to support this.	• The panel agreed that dispersible tablets (already in use as antimalarials) have programmatic and cost advantages over suspensions.• Dispersible amoxicillin was added to the EML.• The shortened regimen was not adopted due to a lack of supporting evidence.
**Caffeine citrate** (Injection and oral solution 20 mg/ml; equivalent to 10 mg caffeine base/ml)	• For treatment of neonatal apnoea: 20 mg/kg loading dose, followed by 5–10 mg/kg daily until resolution of apnoea [1].	• Estimation of the impact of the use of caffeine was limited by a lack of Ghanaian data on the incidence of neonatal apnoea.• Currently there are no suitable products listed in either the IDPI price guide or the WHO sources and prices of medicines.	• The panel noted that some tertiary children's hospitals in Ghana are currently importing and preparing their own caffeine product.• Caffeine citrate was added to the EML with plans to identify a local manufacturer.

### 1. Applicability: Few of the Trials Were Conducted in Africa

The applicability of the evidence base for both zinc sulphate (for diarrhoea) and chlorhexidine 4% solution (for cord care) to Ghana was limited, as the majority of available data came from Asian countries, where the effects could reasonably be expected to be different.

Zinc sulphate is recommended by the WHO as an adjunct to oral rehydration therapy for children with acute diarrhoea [14]. However, of the 44 trials included in the Cochrane review of zinc therapy, only two were conducted in Africa, neither of which demonstrated a clinical benefit [15]. In Asia, zinc appeared to shorten the duration of diarrhoea but with significant heterogeneity in the size of this effect. Sub-group analyses suggests that the effect is largest in children aged greater than 6 months with signs of moderate malnutrition, and as a nutritional intervention this has a logical consistency. It was on this basis that the committee decided to introduce Zinc sulphate in Ghana, where malnutrition in some rural areas is above 30% [16].

### 2. Applicability: The Control Groups Used in Trials Differ from Current Practice

The applicability of the global evidence base for chlorhexidine, and artesunate (for severe malaria), was limited because the control groups used in the primary research were different to current practice in Ghana.

Chlorhexidine was included on the WHO list of priority medicines for application to the umbilical stump to prevent neonatal sepsis [1]. The published evidence evaluating chlorhexidine (available in 2011), was limited to a single large trial from Nepal [17]. This trial had two control interventions: soap and water and dry cord care, whereas current practice in Ghana most commonly involves the application of alcohol: an intervention that has itself never been evaluated. In addition, the intervention in the Nepalese efficacy trial was delivered via an intensive regimen of post-natal home visits, which would be neither feasible nor affordable in Ghana. As a consequence, the committee considered the evidence insufficient to introduce chlorhexidine nationwide at that time.

Similarly, most trials evaluating artesunate have compared it to quinine, but in Ghana, as in many sub-Saharan countries, injectable artemether has become widely popular due to the ease of intra-muscular administration. Only a single trial in adults from Asia has directly compared artesunate with artemether and the result did not reach statistical significance [18].

### 3. Cost and Cost-Effectiveness: Data Not Available

The cost of introducing each medicine was important for the committee, but none of the WHO documents recommending these five medicines included advice or guidance on costs [1],[14],[19]–[21].

Searches of the NHS Economics Evaluations Database found two economic evaluations of artesunate versus quinine [22],[23]. One of these presented a cost-benefit analysis from three African study sites involved in a large efficacy trial [24], and found artesunate to be highly cost-effective in comparison to quinine (an estimated US$123 per additional life saved) [22].

Only one further cost-effectiveness evaluation was available, for zinc sulphate, which had limited applicability to Ghana [25]. This evaluation concluded that the additional costs of zinc sulphate were offset by gains in the mothers' time and productivity through reduced illness duration. From a health providers' perspective, although a course of zinc is relatively cheap (US$0.28), the burden of diarrhoeal disease is such that drug costs may be substantial.

### 4. Feasibility: Limitations with the Current Supply Chain

The committee was concerned about the harmful effects of changing national policy before a reliable supply of a new drug was assured.

Artesunate was recommended as the first-line antimalarial for severe malaria in Africa in a 2011 update to the WHO Malaria Treatment Guidelines [19]. The committee accepted the evidence base provided, and the cost implications of introducing artesunate described above, but were concerned that if they authorised a switch from quinine and artemether (which are widely available and of good manufacturing quality), to artesunate (where the supply may be less reliable and with added threat of fake drugs), this may actually increase mortality from malaria. The committee therefore added artesunate to the Ghana EML but deferred changes to the national malaria guidelines until a reliable supply was established. These feasibility concerns were discussed at a global stakeholder meeting in November 2011 [26].

Reliable international or national suppliers were also limited for dispersible zinc tablets, dispersible amoxicillin tablets, and caffeine citrate [11],[12].

## Project Outcome

Four of the five priority medicines were approved by the expert committee for addition to the Ghana EML: zinc sulphate, artesunate, dispersible amoxicillin, and caffeine citrate. The fifth, chlorhexidine, will be re-considered once the ongoing African effectiveness studies have been published.

## Learning Points

Confident national decisions require understanding and debate of the evidence-base underlying global recommendations, plus additional consideration of national conditions and resources.

To facilitate this, global recommendations should be presented alongside transparent descriptions of the evidence base, allowing policy groups to identify where, when, and how the interventions have been evaluated, and any factors limiting wider applicability.

In addition, for interventions where feasibility and affordability are likely to vary from setting to setting, the WHO could further assist national decision-makers by providing implementation guidance on the assessment of health system implications, training and education requirements, and country level cost analyses.

As many policy questions are relevant across sub-Saharan Africa, and national policy makers are likely to encounter similar problems, we strongly encourage regional collaboration on evidence evaluation, and sharing of information and resources. We hope this paper will encourage further capacity building initiatives, which facilitate and empower countries to make more informed decisions, choosing the interventions that are right for their context, and not implementing unproven interventions.

## Supporting Information

Text S1 **Artesunate evidence summary.**
(PDF)

Text S2 **Artemether evidence summary.**
(PDF)

Text S3 **Zinc evidence summary.**
(PDF)

Text S4 **Chlorhexidine evidence summary.**
(PDF)

Text S5 **Amoxicillin evidence summary.**
(PDF)

Text S6 **Caffeine evidence summary.**
(PDF)
