# Supplementary material for: Integrating Global and National Knowledge to Select Medicines for Children: The Ghana National Drugs Programme
Source: PLoS Med. 2013 May 21;10(5):e1001449. doi: 10.1371/journal.pmed.1001449 (PMC3660441; doi:10.1371/journal.pmed.1001449)
Supplement: Text S1 — Artesunate evidence summary. (PDF) [file pmed.1001449.s001.pdf]

Evidence Summary
For the Ghana Essential Medicines Committee

Title: **Artesunate for treating severe malaria**

Formulation: Artesunate 60mg vial for injection (plus 5% sodium bicarbonate buffer)

Executive Summary

Context: The WHO recently changed its guidelines to recommend artesunate as first line therapy for severe malaria in all settings.
In Ghana, first-line treatment remains as quinine i.v. or artemether i.m.
Artesunate is listed on the Ghana EML, but not the NHIL.

Effects: **Benefits of treating with artesunate instead of quinine**

- Fewer children will die from severe malaria *(high quality evidence)*.

Harms of treating with artesunate instead of quinine

- There is a small increase in the number of children with neurological sequelae at the time of hospital discharge *(high quality evidence)*,
- But there is probably no increase in long-term neurological sequelae *(moderate quality evidence)*.

Feasibility: A reliable supplier needs to be identified
May require significant refresher training of clinical staff

Acceptability: Will require co-ordination with National Malaria Control Programme and Ghana National Health Insurance Authority.

Cost: $ 3 additional cost per patient treated *(moderate quality evidence)*,
$ 429 per additional life saved (using NMCP estimates of mortality)
$ 180,000 additional cost to the Ghana malaria control programme per year

Conclusion: Artesunate is superior to quinine for the treatment of severe malaria.

For consideration: Consider the addition of artesunate to the NHIL.
Consider revision of Ghana Malaria Treatment Guidelines for severe malaria

About this evidence summary
Who prepared this summary? This summary was prepared by Brian Adu Asare & David Sinclair under the supervision of the Ghana Ministry of Health National Drugs Programme and with technical support from the Liverpool School of Tropical Medicine.
Who funded this evidence summary? Training and technical support for the production of this summary was funded by the Bill and Melinda Gates Foundation through the 'Better Medicines for Children Project'; co-ordinated by the World Health Organization, in partnership with the Ghana Ministry of Health.
Declaration of conflicts of interest: None declared

Context

Why should this formulation be considered by the committee?

In 2008 the World Health Organization recommended a change in the first-line treatment of severe malaria in adults in Asia from quinine to artesunate, but there was insufficient evidence at that time to make a similar recommendation in children in Africa. The results of a large multi-centre trial in nine African countries, have now become available, and consequently, in an amendment to the second edition WHO Malaria Treatment Guidelines, artesunate is now recommended as the first-line treatment of choice for severe malaria in all settings (WHO 2010a).

Consequently, the Ghana Standard Treatment Guidelines, and the Ghana Pocketbook for Hospital Care of Children may need updating to reflect this change in international guidelines. The first line treatment in Ghana remains an intravenous infusion or intramuscular injection of quinine. A loading dose of 20 mg/kg is recommended, with subsequent dosing at 10 mg/kg at eight hourly intervals (Ghana STG).

Artesunate is currently listed on the Ghana Essential Medicines List. However it is not currently on the National Health Insurance List of refundable medicines (Ghana EML, Ghana NHIL).

What questions does this evidence summary aim to address?

This evidence summary aims to answer the following questions:

1. **Is artesunate superior to quinine for treating severe malaria in Ghana?**
2. **What would be the public health impact of using artemisinin instead of quinine?**
3. **Is the suggested formulation feasible and acceptable for introduction in Ghana?**
4. **What are the resource implications for this change in policy?**

Effects

Q. Is artesunate superior to quinine for treating severe malaria?

What is severe malaria and how might artesunate work?

Severe malaria occurs when infection with the malaria parasite is complicated by serious failure of the body's major organs. Sometimes severe malaria is associated with coma, which is known as cerebral malaria. Following cerebral malaria a small proportion of children suffer with long-term neurological problems (Jaffar 1997).

Compared to quinine, the artemisinin derivatives have been shown to clear malaria parasites from the blood faster, and to have a broader spectrum of activity (Adjuik 2004). Importantly they are effective against young ring forms of the parasite before they sequester in the microcirculation of vital organs, a major patho-physiological step in the development of severe disease (WHO 2000).

The artemisinin derivatives are generally regarded as safe in humans. Animal studies using very high doses of artemisinin derivatives have demonstrated focal brain stem lesions particularly affecting the auditory pathways, but studies of brain stem function in humans, including audiometry, have failed to show any abnormality following repeated courses (Nosten 2007).

What research evidence is available?

In August 2011 we searched the Cochrane Library and Pubmed for systematic reviews comparing Artesunate with the standard treatment, quinine, for the treatment of severe malaria (see Annex 1 for the detailed search strategy).

We found one Cochrane review, up-to-date to December 2010, and published online in January 2011. Several older non-Cochrane reviews are available but these were published prior to the publication of the large multi-centre trial in African children (Dondorp 2010), and consequently are now out of date (Praygod 2008, Kyu 2009).

What does the research show?

The Cochrane review aimed to summarize the benefits and harms of artesunate compared to quinine for treating severe malaria. Eight randomized controlled trials were included, enrolling 1664 adults and 5765 children.

The benefits of using artesunate instead of quinine:

- Artesunate significantly reduces the risk of death both in adults (RR 0.71, 95% CI 0.58 to 0.86; 1562 participants, five trials, HIGH quality evidence) and children (RR 0.76, 95% CI 0.65 to 0.90; 5765 participants, four trials, *high quality evidence*),
- Artesunate may not clear fever quicker than quinine (the data could not be pooled, *low quality evidence*),
- Artesunate probably clears parasites from the blood quicker than quinine (the data could not be pooled, *moderate quality evidence*),
- Artesunate reduces the risk of hypoglycaemia in both adults (RR 0.36, 95% CI 0.19 to 0.68, 1372 participants, 2 trials, *high quality evidence*), and children (RR 0.58, 95% CI 0.42 to 0.79, 6958 participants, 3 trials, *high quality evidence*).

The harms of using artesunate instead of quinine:

- In children, treatment with artesunate increases the incidence of neurological sequelae at the time of hospital discharge (RR 1.4, 95% CI 1.05 to 1.87, 6151 participants, 3 trials, *high quality evidence*).
- However, these neurological sequelae appear to be transient and there is probably no increase in long term sequelae (RR 1.23, 95% CI 0.74 to 2.03, 4857 participants, 1 trial, *moderate quality evidence*).

About systematic reviews

What is a systematic review? A systematic review seeks to answer a well formulated and specific question by identifying, critically appraising, and summarising the results of all relevant trials, published and unpublished, according to pre-stated and transparent methods.

What is a Cochrane Systematic Review? The Cochrane Collaboration is an international network of more than 28,000 people from over 100 countries. The collaboration is one of the biggest producers of systematic reviews on the effects of healthcare interventions, and Cochrane Systematic Reviews are recognized internationally as the benchmark for high quality information. Over 4,600 reviews have now been published online in *The Cochrane Library*. http://www.thecochranelibrary.com

What about non-Cochrane systematic reviews? Non-Cochrane reviews can be variable in quality. Important predictors of quality are: a broad and exhaustive search strategy, an assessment of the risk of bias of included studies, and freedom from conflicts of interest.

Are the results of the research reliable?

How much confidence can we have in the systematic review methods?

The Cochrane review is well conducted with only minor limitations (see Annex 2). An extensive search was conducted of the CENTRAL, MEDLINE, EMBASE, LILACS and CINAHL databases. It is unlikely that published trials assessing this question have been missed. The authors also searched for unpublished trials by reviewing trial registers and conference proceedings but none were found.

How much confidence can we have in the systematic review results?

The quality of the evidence provided by the Cochrane review has been assessed using the methods developed by the GRADE working group. A summary of the main results of the review, and the quality assessments is shown overleaf in the Summary of Findings table.

The evidence for a reduction in mortality is considered to be of high quality, meaning that we can be confident that this result is accurate, and further research is unlikely to change the estimate of effect.

The absolute benefit in children appears lower than in adults. This is due to lower mortality seen in the trials recruiting children. This lower mortality could be explained by: a lower threshold for classifying malaria in children as 'severe'; higher levels of acquired immunity among children (providing some protection against death); or higher efficacy of quinine in Africa. The mortality from severe malaria in children may be higher outside of the study context.

Can the results of the research be applied to Ghana?

The majority of data in children is from a large multi-centred trial, conducted between 2005 and 2010 (Dondorp 2010). This included study sites in Ghana, the Gambia, DRC, Kenya, Mozambique, Nigeria, Rwanda, Tanzania, and Uganda, and the benefits on mortality appear consistent across all settings.

The median age of children in this study was 2.8 years (inter-quartile range 1.6 to 4.2).

The standard doses of quinine and artesunate were used: Quinine 20 mg salt/kg infused over 4 hours in 5-10ml/kg 5% dextrose, followed by 10 mg salt/kg every 8 hours; Artesunate 2.4 mg/kg on admission, at 12hrs, 24 hrs, and then daily until oral therapy tolerated.

Once oral therapy was tolerated a complete 3-day course of Artemisinin-based combination therapy was given to both groups.

About quality of evidence (GRADE)

The GRADE system considers 'quality' to be a judgment of the extent to which we can be confident that the estimates of effect are correct. The level of 'quality' is judged on a 4-point scale. Evidence from randomized controlled studies is initially graded as HIGH and downgraded by one, two or three levels after full consideration of : any limitations in the design of the studies, the directness (or applicability) of the evidence, and the consistency and precision of the results.

- **High:** Further research is very unlikely to change our confidence in the estimate of effect.
- **Moderate:** Further research is likely to have an important impact on our confidence in the estimate of effect and may change the estimate.
- **Low:** Further research is very likely to have an important impact on our confidence in the estimate of effect and is likely to change the estimate
- **Very low:** We are very uncertain about the estimate.

Summary of findings table

Artesunate compared to Quinine for treating severe malaria

Patient or population: adults and children with severe malaria
Settings: low and middle income countries

Outcomes	Illustrative comparative risks* (95% CI)		Relative effect (95% CI)	No of Participants (studies)	Quality of the evidence (GRADE)	Comments
	Assumed risk	Corresponding risk				
	Quinine	Artesunate				
**Death**	**Adults**					
	**241 per 1000**	**171 per 1000** (140 to 207)	**RR 0.71** (0.58 to 0.86)	1562 (5 studies)	**high**[1]	
	**Children**					
	**109 per 1000**	**83 per 1000** (71 to 98)	**RR 0.76** (0.65 to 0.9)	5765 (4 studies)	**high**[2]	
**Neurological sequelae - At discharge**	**Adults**					
	**5 per 1000**	**11 per 1000** (3 to 41)	**RR 2.12** (0.55 to 8.17)	1190 (1 study)	**moderate**[3]	
	**Children**					
	**25 per 1000**	**35 per 1000** (26 to 47)	**RR 1.4** (1.05 to 1.87)	6151 (3 studies)	**high**	
**Neurological sequelae - At day 28**	**Adults**					Not measured
	**-**	**-**	**-**	(0 studies)	**-**	
	**Children**					
	**11 per 1000**	**14 per 1000** (8 to 22)	**RR 1.23** (0.74 to 2.03)	4857 (1 study)	**moderate**[3]	
**Episodes of hypoglycaemia**	**Adults**					
	**47 per 1000**	**17 per 1000** (9 to 32)	**RR 0.36** (0.19 to 0.68)	1372 (2 studies)	**high**	
	**Children**					
	**30 per 1000**	**17 per 1000** (13 to 24)	**RR 0.58** (0.42 to 0.79)	6958 (3 studies)	**high**	

*The basis for the **assumed risk** is the risk of death in the groups treated with quinine in the included trials. Under these trial conditions, the risk of death may be underestimated.
The **corresponding risk** (and its 95% confidence interval) is based on the assumed risk in the comparison group and the **relative effect** of the intervention (and its 95% CI).
CI: Confidence interval; **RR:** Risk ratio;

[1] Five studies included adults: Three small studies were conducted in Vietnam and Thailand, and one large multicentre trial had study sites in Bangladesh, India, Myanmar and Indoneisa. there was no reason to grade down for study limitations, imprecision, inconsistency, indirectness, or publication bias.
[2] Four studies included children: The majority of child data is from one large multicentre trial with sites in Ghana, Gambia, DRC, Nigeria, Mozambique, Rwanda, Tanzania, Kenya and Uganda. there was no reason to grade down for study limitations, imprecision, inconsistency, indirectness, or publication bias.
[3] This result is not statistically significant, but the 95% CI is wide and includes the possibility of a clinically important harm with artesunate. Downgraded for imprecision by 1 level.

Q2. What is the potential public health impact of applying the results to Ghana?

In 2009, the Ghana National Malaria Control Programme records that 61,462 were diagnosed with severe malaria, of whom 1,906 died (NMCP 2009). This mortality rate in children (3.1%) is much lower than was seen in the African trials (10.9%). The reasons for this disparity are unclear; but possibilities include under reporting of severe malaria deaths, or over diagnosis of severe malaria (including less severe cases).

Applying the relative risk reduction to this lower mortality rate suggest that using artesunate instead of quinine in Ghana would save 7 lives per 1,000 children treated, with a Number Needed to Treat to prevent one death (NNT) of 143. In total this would prevent 430 childhood deaths per year.

Using the mortality data from the African trials, artesunate would save 26 lives per 1,000 children treated, with a NNT of 38.

Q3. Is the current formulation suitable for introduction to Ghana?

Description of the formulation

**Route of administration:**	Intravenous or intramuscular
**Dosing schedule:**	2.4mg/kg on admission, at 12 h and 24h then daily until able to tolerate oral
**Additional requirements:**	No specialised monitoring is required
**Storage:**	As dry powder for reconstitution with 5% sodium bicarbonate, and saline
**Stability:**	?
**Transport:**	?

Is the introduction of this formulation feasible?

**Locally available manufacturers:**	None
**Ghana FDB Registration:**	None
**International manufacturers:**	5: India (Esco, IPCA, Macleods, Neon), China (Guilin)
**Suggested level of prescribing:**	D, C, B2,
**Educational requirements:**	May require significant refresher training for clinical staff
**System requirements:**	None
**Any other concerns:**	Is there a reliable supplier of adequate quantities?

Will the introduction of this formulation be acceptable to all stakeholders?

**Appropriateness of formulation:**	Suitable for administration to children and adults
**Additional Stakeholders:**	Ghana National Malaria Control Programme, The National Health Insurance Authority
**National Guidelines:**	The Standard Treatment Guidelines, and Ghana Pocketbook for Paediatric care would need updating
**International Guidelines**	This change would bring Ghana in-line with current international guidelines (WHO 2010)

Q4. What are the resource implications?

What does this formulation cost?

	Formulation	Median	Minimum	Maximum
**IDPI Price Guide:**	None listed		-	-
**WHO Sources and prices 2nd edition:**	60mg vial with buffer	$ 0.8	$ 0.3	$ 0.97

Is it cost-effective?

We searched the Economic Evaluation Database within the Cochrane library for cost-effectiveness analyses of artesunate compared to quinine. We found one from Africa (Lubell 2011), and one from Asia (Lubell 2009).

The African study was conducted during the same large multi-centre trial on which the estimates of effect are based (Dondorp 2010). Costs were assessed at four of the study sites; Tanzania (2), Uganda, and Nigeria. An assessment of the methods of this study is given in Annex 4.

A service providers perspective was taken and the economic benefits to society of each additional life saved were not assessed. The incremental cost of treating with artesunate instead of quinine was $3 per patient.

Based on the trial data, with a NNT of 41, this gives an incremental cost of $123 per additional life saved. Applying these data to the Ghanaian estimates of prevalence and mortality would give $498 per additional life saved, and a total annual incremental cost of $184,000 per year.

Artesunate compared to Quinine for treating severe malaria

Patient or population: adults and children with severe malaria
Settings: low and middle income countries

Resource	Costs		Difference	No of Participants (studies)	Quality of the evidence (GRADE)	Comments
	Quinine	Artesunate				
Trial drug	$ 1.3	$ 3.3	**+ $ 2.0**	2300 (1)	High[1,2]	
Additional drugs	$ 3.0	$ 2.9	**- $ 0.1**	2300 (1)	High[1,2]	
Fluids	$ 13.5	$ 12.5	**- $ 1.0**	2300 (1)	Moderate[1,3]	Range (-$4.4 to +$1.0)
Laboratories	$ 11.6	$ 11.5	**- $ 0.1**	2300 (1)	High[1,2]	
Hotel	$ 34.1	$ 36.3	**+ $ 2.2**	2300 (1)	Moderate[1,3]	Range ($0.6 to +$17.3)
**Total cost per treatment episode**	**$ 63.5**	**$ 66.5**	**+ $ 3.0**	2300 (1)	Moderate[1,3]	
**Incremental cost per additional death avoided**	**-**	**$ 123.0**				Based on NNT = 41[4]
**Incremental cost per additional death avoided**	**-**	**$ 429.0**				Based on NNT = 143[5]

*$1 = 1.7 Ghanaian Cedis

[1] This data is based on a cost-effectiveness analysis at 4 study sites in the Aquamat trial: Tanzania (2), Uganda & Nigeria (Dondorp 2010)
[2] There was no reason to downgrade for study limitations, consistency, directness or precision.
[3] Downgraded for consistency due to high variability between study sites
[4] This NNT is taken from the Dondorp 2010 and was used in the cost effectiveness analysis by Lubell 2011
[5] This NNT is calculated from the Ghana NMCP 2009 data

About the NHS Economic Evaluations Database within the Cochrane Library
As healthcare resources are finite, information about both costs and effects are essential to making evidence-based decisions about competing healthcare interventions. But information about cost-effectiveness can be difficult to identify, appraise and interpret.
The NHS Economic Evaluation Database (EED) assists decision-makers by systematically identifying economic evaluations from around the world, appraising their quality, and highlighting their relative strengths and weaknesses.
The NHS Economic Evaluations Database is produced by the Centre for Reviews and Dissemination (CRD) at the University of York, UK.

References

- Ghana National Malaria Control Programme Annual Report 2009.
- World Health Organization. Roll Back Malaria Dept. Guidelines for the treatment of malaria; Second edition. Geneva: World Health Organization, 2010.
- Ghana Ministry of Health. Ghana Standard Treatment Guidelines. Sixth edition. 2010. Ghana National Drugs Programme. Available at: http://ghndp.org
- Ghana Ministry of Health. Ghana Essential Medicines List, Sixth edition. 2010. Ghana National Drugs Programme. Available at: http://ghndp.org
- Jaffar S, Van Hensbroek M, Palmer A, Schneider G, Greenwood B. Predictors of a fatal outcome following childhood cerebral malaria. *American Journal of Tropical Medicine and Hygiene* 1997;**57(1)**: 20–4.
- Adjuik M, Babiker A, Garner P, Olliaro P, Taylor W, White N, International Artemisinin Study Group. Artesunate combinations for treatment of malaria: meta-analysis. *Lancet* 2004;**363**(9402): 9–17.
- WHO. Severe and complicated malaria. *Transactions of the Royal Society of Tropical Medicine and Hygiene* 2000;**94**(suppl 1):1–90.
- Nosten F, White NJ. Artemisinin-based combination treatment of falciparum malaria. American Journal of Tropical Medicine and Hygiene 2007;77 (6 Suppl):181-92.
- Kissinger E, Hien T, Hung N, Nam N, Tuyen N, Dinh B, et al. Clinical and neurophysiological study of the effects of multiple doses of artemisinin on brain-stem function in Vietnamese patients. *American Journal of Tropical Medicine and Hygiene* 2000;**63(1-2)**: 48–55.
- Sinclair D, Donegan S, Lalloo DG. Artesunate versus quinine for treating severe malaria. *Cochrane Database of Systematic Reviews* 2011, Issue 3. Art. No.: CD005967. DOI: 10.1002/14651858.CD005967.pub3.
- Praygod G, de Frey A, Eisenhut M. Artemisinin derivatives versus quinine in treating severe malaria in children: a systematic review. Malaria Journal. 2008; 7:210
- Kyu HH, Fernandez E. Artemisinin derivatives versus quinine for cerebral malaria in African children: a systematic review. *Bulletin of the World Health Organization* 2009;**87**:896–904.
- Dondorp A, Nosten F, Stepniewska K, Day N, White N. Artesunate versus quinine for treatment of severe falciparum malaria: a randomised trial. Lancet 2005;366(9487):717-25.
- Dondorp AM, Fanello CI, Hendriksen IC, Gomes E, Seni A, Chhaganlal KD et al. Artesunate versus quinine in the treatment of severe falciparum malaria in African children (AQUAMAT): an open-label, randomised trial. Lancet 2010;376(9753):1647-57.
- Lubell Y, Riewpaiboon A, Dondorp AM, von Seidlein L, Mokuolu OA, Nansumba M et al. Cost-effectiveness of parenteral artesunate for treating children with severe malaria in sub-Saharan Africa. Bulletin of the World Health Organisation. 2011;89:504-12.
- Lubell Y, Yeung S, Dondorp AM, Day NP, Nosten F, Tjitra E et al. Cost-effectiveness of artesunate for the treatment of severe malaria. Tropical Medicine and International Health. 2009; 14(3): 332–37.

Annex 1. Detailed search strategy and results

**Set**		**Cochrane**	**PubMed**
1		artesunate	artesunate [Title/Abstract]
2		malaria	severe malaria [Title/Abstract]
3		1 AND 2	1 AND 2
4			limit 3 to reviews or meta-analyses
**Search results**		**Cochrane**	**PubMed**
Hits		10	29
Included		1	1 (Cochrane review)
Excluded		9	
Reason for exclusion	Topic not relevant to this summary	9	13
	Not a systematic review		
	More complete reviews are available		16
Additional reviews identified through reference lists		-	-

Annex 2. AMSTAR assessment of the systematic review

Review reference: Sinclair D, Donegan S, Lalloo DG. Artesunate versus quinine for treating severe malaria. *Cochrane Database of Systematic Reviews* 2011, Issue 3. Art. No.: CD005967. DOI: 10.1002/14651858.CD005967.pub3.

**1. Was an 'a priori' design provided?**<br>The research question and inclusion criteria should be established before the conduct of the review.	☑ Yes<br>☐ No<br>☐ Can't answer<br>☐ Not applicable
**2. Was there duplicate study selection and data extraction?**<br>There should be at least two independent data extractors and a consensus procedure for disagreements should be in place.	☑ Yes<br>☐ No<br>☐ Can't answer<br>☐ Not applicable
**3. Was a comprehensive literature search performed?**<br>At least two electronic sources should be searched. The report must include years and databases used (e.g. Central, EMBASE, and MEDLINE). Key words and/or MESH terms must be stated and where feasible the search strategy should be provided. All searches should be supplemented by consulting current contents, reviews, textbooks, specialized registers, or experts in the particular field of study, and by reviewing the references in the studies found.	☑ Yes<br>☐ No<br>☐ Can't answer<br>☐ Not applicable
**4. Was the status of publication (i.e. grey literature) used as an inclusion criterion?**<br>The authors should state that they searched for reports regardless of their publication type. The authors should state whether or not they excluded any reports (from the systematic review), based on their publication status, language etc.	☐ Yes<br>☑ No<br>☐ Can't answer<br>☐ Not applicable
**5. Was a list of studies (included and excluded) provided?**<br>A list of included and excluded studies should be provided.	☑ Yes<br>☐ No<br>☐ Can't answer<br>☐ Not applicable
**6. Were the characteristics of the included studies provided?**<br>In an aggregated form such as a table, data from the original studies should be provided on the participants, interventions and outcomes. The ranges of characteristics in all the studies analyzed e.g. age, race, sex, relevant socioeconomic data, disease status, duration, severity, or other diseases should be reported.	☑ Yes<br>☐ No<br>☐ Can't answer<br>☐ Not applicable
**7. Was the scientific quality of the included studies assessed and documented?**<br>'A priori' methods of assessment should be provided (e.g., for effectiveness studies if the author(s) chose to include only randomized, double-blind, placebo controlled studies, or allocation concealment as inclusion criteria); for other types of studies alternative items will be relevant.	☑ Yes<br>☐ No<br>☐ Can't answer<br>☐ Not applicable
**8. Was the scientific quality of the included studies used appropriately in formulating conclusions?**<br>The results of the methodological rigor and scientific quality should be considered in the analysis and the conclusions of the review, and explicitly stated in formulating recommendations.	☑ Yes<br>☐ No<br>☐ Can't answer<br>☐ Not applicable
**9. Were the methods used to combine the findings of studies appropriate?**<br>For the pooled results, a test should be done to ensure the studies were combinable, to assess their homogeneity (i.e. Chi-squared test for homogeneity, I^2). If heterogeneity exists a random effects model should be used and/or the clinical appropriateness of combining should be taken into consideration (i.e. is it sensible to combine?).	☑ Yes<br>☐ No<br>☐ Can't answer<br>☐ Not applicable
**10. Was the likelihood of publication bias assessed?**<br>An assessment of publication bias should include a combination of graphical aids (e.g., funnel plot, other available tests) and/or statistical tests (e.g., Egger regression test).	☐ Yes<br>☐ No<br>☑ Can't answer<br>☐ Not applicable
**11. Was the conflict of interest stated?**<br>Potential sources of support should be clearly acknowledged in both the systematic review and the included studies.	☑ Yes<br>☐ No<br>☐ Can't answer<br>☐ Not applicable

For information on the AMSTAR tool see: Shea B, Grimshaw J, Wells G, Boers M, Andersson N, Hamel C, et al. Development of AMSTAR: a measurement tool to assess the methodological quality of systematic reviews. BMC Medical Research Methodology. 2007;7(1):10.

Annex 3. Assessment of the local applicability of the systematic review

Review reference:

1. Were the studies included in this systematic review conducted in settings similar to Ghana, or were the findings consistent across settings and time periods?

The large African study included a study site in Ghana.

The findings are consistent across age groups and countries

2. Are there important differences in on-the-ground realities and constraints in Ghana that might substantially alter the feasibility and acceptability of this drug/formulation?

Possibly. There are concerns about the availability of adequate quantities of a good quality product

3. Are there important differences in health system arrangements that may mean this drug/formulation could not work in the same way?

No. But a change in the national guidelines may require considerable refresher training of clinical staff.

4. Are there important differences in the baseline conditions that might yield different absolute effects even if the relative effectiveness was the same?

The best available data on mortality in Ghana is from the Ghana National malaria programme. This suggests that mortality is even lower in Ghana than was seen in the trial, which would result in lower absolute benefit.

5. What insights can be drawn about options, implementation, and monitoring and evaluation?

Unclear

For further information on the SUPPORT tool used for this assessment see: Lavis JN, Oxman AD, Souza NM, Lewin S, Gruen RL, Fretheim A. SUPPORT Tools for evidence-informed health Policymaking (STP) 9: Assessing the applicability of the findings of a systematic review. Health Research Policy and Systems. 2009; 7 (Suppl 1):S9

Annex 4. Assessment of the economic analysis

Cost effectiveness reference:		Comment
1. Is the study population clearly described?	☑ Yes ☐ No ☐ Unclear	Same as Dondorp 2010
2. Are competing alternatives clearly described?	☑ Yes ☐ No ☐ Unclear	Treatment with artesuntate versus treatment with quinine
3. Is a well-defined research question posed in answerable form?	☑ Yes ☐ No ☐ Unclear	
4. Is the economic study design appropriate to the stated objective?	☑ Yes ☐ No ☐ Unclear	
5. Is the chosen time horizon appropriate to include relevant costs and consequences?	☑ Yes ☐ No ☐ Unclear	Severe malaria is an acute condition, so immediate inpatient costs are most relevent
6. Is the actual perspective chosen appropriate?	☑ Yes ☐ No ☐ Unclear	A providers perspective is taken
7. Are all important and relevant costs for each alternative identified?	☑ Yes ☐ No ☐ Unclear	All costs to the health service: drug, staff and bed costs, have been evaluated
8. Are all costs measured appropriately in physical units?	☑ Yes ☐ No ☐ Unclear	
9. Are costs valued appropriately?	☑ Yes ☐ No ☐ Unclear	
10. Are all important and relevant outcomes for each alternative identified?	☑ Yes ☐ No ☐ Unclear	
11. Are all outcomes measured appropriately?	☑ Yes ☐ No ☐ Unclear	
12. Are outcomes valued appropriately?	☐ Yes ☐ No ☑ Unclear	This is a cost-benefit analysis.
13. Is an incremental analysis of costs and outcomes of alternatives performed?	☑ Yes ☐ No ☐ Unclear	
14. Are all future costs and outcomes discounted appropriately?	☐ Yes ☐ No ☑ Unclear	Not relevent
15. Are all important variables, whose values are uncertain, appropriately subjected to sensitivity analysis?	☐ Yes ☐ No ☐ Unclear	
16. Do the conclusions follow from the data reported?	☑ Yes ☐ No ☐ Unclear	
17. Does the study discuss the generalizability of the results to other settings and patient/client groups?	☑ Yes ☐ No ☐ Unclear	
18. Does the article indicate that there is no potential conflict of interest of study researcher(s) and funder(s)?	☑ Yes ☐ No ☐ Unclear	
19. Are ethical and distributional issues discussed appropriately?	☐ Yes ☐ No ☑ Unclear	

For further information on the CHEC-list used for this assessment see: Evers S, Goossens M, de Vet H, van Tulder M, Ament A: Criteria list for assessment of methodological quality of economic evaluations: Consensus on Health Economic Criteria. International Journal of Technology Assessment in Health Care. 2005; 21:240-5.
