# Supplementary material for: Integrating Global and National Knowledge to Select Medicines for Children: The Ghana National Drugs Programme
Source: PLoS Med. 2013 May 21;10(5):e1001449. doi: 10.1371/journal.pmed.1001449 (PMC3660441; doi:10.1371/journal.pmed.1001449)
Supplement: Text S2 — Artemether evidence summary. (PDF) [file pmed.1001449.s002.pdf]

Evidence Summary
For the Ghana Essential Medicines Committee

Title: **Artemether for treating severe malaria**

Formulation: Injection Artemether 40mg / 80mg (for intramuscular use)

Executive Summary

Context: Artesunate has been shown to reduce mortality from severe malaria compared to quinine and is now recommended by the World Health Organization as the first-line treatment in all settings.
In light of this evidence it is important to re-examine the role of artemether, which is widely available and widely used in Ghana.
Should Artemether remain as a first-line treatment option, or should it become a second line option where artesunate is unavailable?

Effects: **Benefits of treating with artesunate instead of artemether**

- Artesunate may reduce mortality compared to artemether (*low quality evidence*)

Benefits of treating with artemether instead of quinine

- Artemether probably does not reduce mortality compared to quinine (*moderate quality evidence*)

Feasibility: Restriction of use of artemether would require refresher training of prescribers, and a reliable supply of artesunate.

Acceptability: Would require co-ordination with the National Malaria Control Programme.

Cost: Cost analyses of artemether vs. quinine, or artemether vs. artesunate were not available.

Conclusion: Current evidence suggests artemether is probably equivalent in efficacy to quinine.

For consideration: Consider promotion of artesunate to first-line treatment for severe malaria in Ghana?
Consider withdrawal of artemether from EML/NHIL?

About this evidence summary

Who prepared this summary? This summary was prepared by Daniel Buabin & Nii Obadai Mensah with technical support from the Liverpool School of Tropical Medicine.

Who funded this evidence summary? Training and technical support for the production of this summary was funded by the Bill and Melinda Gates Foundation through the 'Better Medicines for Children Project', co-ordinated by the World Health Organization, in partnership with the Ghana Ministry of Health National Drugs Programme.
Declaration of conflicts of interest: None declared

Context

Why should this drug/formulation be considered by the committee?

In 2008 the World Health Organization recommended a change in the first-line treatment of severe malaria in adults in Asia from quinine to artesunate, but there was insufficient evidence at that time to make a similar recommendation in children in Africa.

In 2010, the results of a large multi-centre trial in 9 African countries were published, which confirmed the superiority of artesunate over quinine (Dondorp 2010). A separate evidence summary of artesunate in severe malaria has been compiled. Consequently, in an amendment to the second edition WHO Malaria Treatment Guidelines, artesunate is now recommended as the first-line treatment of choice for severe malaria in all settings (WHO 2010).

In light of this evidence it is important to re-examine the role of artemether, which is widely available and widely used in Ghana. Should artemether remain as a first-line treatment option, or should it be restricted to situations where artesunate is unavailable.

Injectable artemether is currently listed on the Ghana Essentials Medicine list and the National Health Insurance List (Ghana EML, Ghana NHIL). It is recommended as an alternative to the current first-line treatment, quinine (Ghana STG).

What questions does this evidence summary aim to address?

This evidence summary aims to answer the following questions:

1. **Is artesunate superior to artemether for treating severe malaria in Ghana?**
2. **Is a change in policy from artemether to artesunate acceptable and feasible in Ghana?**
3. **What would be the public health benefits of this change?**
4. **What are the resource implications?**

Effects

Q1. Is artesunate superior to artemether for treating severe malaria?

What is severe malaria and how might artemether work?

Severe malaria occurs when infection with the malaria parasite is complicated by serious failure of the body's major organs. Sometimes severe malaria is associated with coma, which is known as cerebral malaria.

Intravenous or intramuscular quinine has been the drug of choice to treat severe or cerebral malaria in most malaria endemic countries. Intramuscular medication can be easier to administer, especially in young children or patients with poor peripheral perfusion, but absorption can be less reliable.

Artemether is administered by intramuscular injection and its absorption is erratic, particularly in severely ill patients. As a consequence recent research attention has concentrated on artesunate which can also be given intravenously (Hien 2004). The recommended dose of artemether is 3.2mg/kg body weight IM given on admission, then 1.6mg/kg daily. Unlike quinine it does not require monitoring of cardiac function or glucose levels.

What research evidence is available?

In August 2011 we searched the Cochrane Library and PubMed for systematic reviews comparing artemether with either artesunate or quinine for the treatment of severe malaria (see Annex 1 for the detailed search strategy).

We did not find any reviews comparing artemether with artesunate so we expanded our search to look for randomised controlled trials for this comparison. We only found one randomised controlled trial (Phu 2010).

The only Cochrane review which assesses artemether is now out of date and no longer updated due to the shift in interest towards artesunate (McIntosh 2001). Three more recent non-Cochrane reviews were retrieved (Matthew 2010, Kyu 2009 and Praygod 2008), which compared the artemisinin derivatives in general (including artemether) to quinine. Older reviews were available but were not reviewed due to being out of date.

What does the research show?

Phu 2010 is the only published trial to directly compare artesunate and artemether in the management of severe malaria. This trial was conducted in adults in Vietnam and was stopped early due to a declining incidence of severe malaria. Consequently the result did not reach statistical significance.

The benefits of using artesunate instead of artemether:

- ❖ Artesunate may reduce mortality compared to artemether (RR 0.54, 95%CI 0.28-1.02, 370 participants, 1 trial, *low quality evidence*).
- ❖ Artesunate probably does not clear parasites quicker than artemether (370 participants, 1 trial, *moderate quality evidence*).
- ❖ Artesunate probably does not clear parasites quicker than artemether (370 participants, 1 trial, *moderate quality evidence*).

Praygod 2008 presents the most complete meta-analysis of artemether versus quinine but data is only available for the primary outcome of mortality. Matthew 2010 only searched Medline, and Kyu 2009 limited the search to trials of cerebral malaria. Both reviews found similar results to Praygod 2008 which is presented here. Nine randomized controlled trials of Artemether were included, enrolling 1258 participants.

The benefits of using artemether instead of quinine:

- ❖ Artemether IM probably does not reduce mortality compared to quinine (RR 0.94 95% CI 0.74 to 1.19, 1258 participants, 9 trials, *moderate quality evidence*).

About systematic reviews

What is a systematic review? A systematic review seeks to answer a well formulated and specific question by identifying, critically appraising, and summarising the results of <u>all</u> relevant trials, published and unpublished, according to pre-stated and transparent methods.

What is a Cochrane Systematic Review? The Cochrane Collaboration is an international network of more than 28,000 people from over 100 countries. The collaboration is one of the biggest producers of systematic reviews on the effects of healthcare interventions, and Cochrane Systematic Reviews are recognized internationally as the benchmark for high quality information. Over 4,600 reviews have now been published online in *The Cochrane Library*. http://www.thecochranelibrary.com

What about non-Cochrane systematic reviews? Non-Cochrane reviews can be variable in quality. Important predictors of quality are: a broad and exhaustive search strategy, an assessment of the risk of bias of included studies, and freedom from conflicts of interest.

Are the results of the research reliable?

How much confidence can we have in the research methods?

The single RCT (Phu 2010) was assessed using the Cochrane tool for assessing the risk of bias (see Annex 2). It is generally well conducted and is considered to be at low risk of bias.

The systematic review (Praygod 2008) was assessed using the AMSTAR tool and has several limitations (see Annex 3). However a comprehensive literature search was undertaken and this represents the most complete assessment of artemether available.

How much confidence can we have in the research results?

The quality of the evidence provided by both the RCT and the systematic review has been assessed using the methods developed by the GRADE working group. A summary of the main results, and the quality assessments, is shown overleaf in the Summary of Findings tables.

The evidence that artemether is inferior to artesunate at reducing mortality from severe malaria, is considered to be of low quality, meaning that further research is very likely to change the estimate of effect. The evidence was downgraded from high to low due to concerns about:

- The 'directness' of the evidence: with this single trial being conducted in Asian adults.
- The 'precision' of the estimate which did not quite reach statistical significance.

Can the results of the research be applied to Ghana?

The single trial of artesunate versus Artemether was conducted in Asian adults, and so is not easily applied to Ghana where most severe malaria occurs in children. However, this finding that artemether may be inferior to artesunate is consistent with evidence from African countries that artemether is probably equivalent to quinine, and quinine is inferior to artesunate.

About quality of evidence (GRADE)

The GRADE system considers 'quality' to be a judgment of the extent to which we can be confident that the estimates of effect are correct. The level of 'quality' is judged on a 4-point scale. Evidence from randomized controlled studies is initially graded as HIGH and downgraded by one, two or three levels after full consideration of : any limitations in the design of the studies, the directness (or applicability) of the evidence, and the consistency and precision of the results.

High: Further research is very unlikely to change our confidence in the estimate of effect.
Moderate: Further research is likely to have an important impact on our confidence in the estimate of effect and may change the estimate.
Low: Further research is very likely to have an important impact on our confidence in the estimate of effect and is likely to change the estimate
Very low: We are very uncertain about the estimate.

Summary of findings table 1

Artesunate compared to Artemether for treating severe malaria

Patient or population: Children with severe malaria
Settings: Low and middle income countries

Outcomes	Illustrative comparative risks* (95% CI)		Relative effect (95% CI)	No of Participants (studies)	Quality of the evidence (GRADE)	Comments
	Assumed risk **Artemether**	Corresponding risk **Artesunate**				
**Death**	**130 per 1000**	**70 per 1000** (36 to 133)	**RR 0.54** (0.28 to 1.02)	370 Participants (1 study)	**low** [1,2,3,4,]	
**Parasite clearance**	The median time to parasite clearance in the control group was **72 hours**	The median time to parasite clearance in the intervention group was **72 hours**		370 Participants (1 study)	**moderate** [1,2,3]	P = 0.97
**Fever clearance**	The median time to fever clearance in the control group was **108 hours**	The median time to fever clearance in the intervention group was **108 hours**		370 Participants (1 study)	**moderate** [1,2,3]	P = 0.27
**Coma recovery time**	The median time to coma recovery in the control group was **60 hours**	The median time to coma recovery in the intervention group was **72 hours**		370 Participants (1 study)	**moderate** [1,2,3]	P = 0.11
**Adverse events**				370 Participants (1 study)	**low** [1,2,3,5,6]	

*The basis for the **assumed risk** is the risk of death in the groups treated with artemether in the included trial.
The **corresponding risk** (and its 95% confidence interval) is based on the assumed risk in the comparison group and the **relative effect** of the intervention (and its 95% CI).
CI: Confidence interval; **RR:** Risk ratio;

[1] Only one randomized controlled study has directly compared artesunate with artemether.
[2] There was no reason to downgrade for study limitations.
[3] Downgraded by 1 for directness as this is a single study and the result may not be reliably generalised to other settings (Africa) or populations (Children).
[4] Downgraded by 1 for precision as the result did not reach statistical significance.
[5] Hypoglycaemia occurred in 3 patients treated with artesunate and 5 with artemether. There were no local abscesses, urticaria or rashes and no severe unexpected adverse events'
[6] Downgraded by 1 for precision as the trial is underpowered to exclude important differences in adverse events.

Summary of findings table 2

Artemether compared to Quinine for treating severe malaria

Patient or population: Children with severe malaria
Settings: Low and middle income countries

Outcomes	Illustrative comparative risks* (95% CI)		Relative effect (95% CI)	No of Participants (studies)	Quality of the evidence (GRADE)	Comments
	Assumed risk	Corresponding risk				
	**Quinine**	**Artemether**				
**Death**	**160 per 1000**	**150 per 1000** (118 to 190)	**RR 0.94** (0.74 to 1.19)	1258 (9 studies)	**moderate** [1,2,3,4,5]	

*The basis for the **assumed risk** is the risk of death in the groups treated with quinine in the included trials. Under these trial conditions, the risk of death may be underestimated.
The **corresponding risk** (and its 95% confidence interval) is based on the assumed risk in the comparison group and the **relative effect** of the intervention (and its 95% CI).
CI: Confidence interval; **RR:** Risk ratio;

Footnotes
[1] These data are taken from Praygod 2008 which presented the most complete meta-analysis of this comparison.
[2] There was no reason to downgrade for study limitations: Four studies described adequate randomisation and allocation concealment (Murphy 1996, Van Hensbroek 1996, Taylor 1998, Olumese 1999) and analysis of these four results alone did not significantly change the overall effect estimate.
[3] There was no reason to downgrade for inconsistency: All confidence intervals overlapped and none found a statistically significant difference.
[4] No reason to downgrade for directness: 8 of the trials were in Africa and all studies were conducted in children, ranging from 3 months to 15 years.
[5] Downgraded by 1 for precision: The 95% CI are wide and include the possibility of both a clinically important benefit or harm with artesunate.

Q2. What is the potential public health impact of applying the results to Ghana?

The available evidence suggest that artemether is probably equivalent to quinine, and that its use should be restricted to situations where artesunate is unavailable. An assessment of the public health benefit of using artemether is therefore inappropriate.

Q3. Will a change in policy to artesunate be feasible and acceptable in Ghana?

Description of the formulation

	Artemether	Artesunate
**Route of administration:**	Intramuscular	Intravenous or intramuscular
**Dosing schedule:**	3.2mg/kg on admission then 1.6mg/kg daily until oral therapy tolerated	2.4mg/kg on admission, at 12 h and 24h then daily until able to tolerate oral
**Additional requirements:**	No specialised monitoring is required	No specialised monitoring is required
**Storage:**	Cool dry place with Temperature not exceeding 25^{0} C	As dry powder for reconstitution with 5% sodium bicarbonate, and saline
**Stability:**	?	?
**Transport:**	-	-

Will the restriction of this formulation be acceptable to all stakeholders?

**Additional Stakeholders:**	The Ghana National Malaria Control Programme<br>Clinicians.<br>Local artemether manufacturers.
**National Guidelines:**	National guidelines would need revision. Artemether is currently listed as a first-line alternative to quinine.
**International Guidelines:**	The WHO recommends intravenous artesunate as the first line treatment for severe malaria.<br>The WHO recommend that artemether is only used when artesunate or quinine are unavailable due to its erratic absorption.

Q4. What are the resource implications?

What does this formulation cost?

	Formulation	Median	Minimum	Maximum
**IDPI Price Guide:**	None listed		-	-
**WHO Sources and prices 2nd edition:**	60mg vial with buffer	$ 0.8	$ 0.3	$ 0.97

Is it cost-effective?

We searched the Economic Evaluation Database within the Cochrane library for cost-effectiveness analyses of artemether but none were found.

About the NHS Economic Evaluations Database within the Cochrane Library

As healthcare resources are finite, information about both costs and effects are essential to making evidence-based decisions about competing healthcare interventions. But information about cost-effectiveness can be difficult to identify, appraise and interpret.
The NHS Economic Evaluation Database (EED) assists decision-makers by systematically identifying economic evaluations from around the world, appraising their quality, and highlighting their relative strengths and weaknesses.
The NHS Economic Evaluations Database is produced by the Centre for Reviews and Dissemination (CRD) at the University of York, UK.

References

- Dondorp AM, Fanello CI, Hendriksen IC, Gomes E, Seni A, Chhaganlal KD et al. Artesunate versus quinine in the treatment of severe falciparum malaria in African children (AQUAMAT): an open-label, randomised trial. Lancet 2010;376(9753):1647-57.
- World Health Organization. Roll Back Malaria Dept. Guidelines for the treatment of malaria; Second edition. Geneva: World Health Organization, 2010.
- Ghana Ministry of Health. Ghana Essential Medicines List, Sixth edition. 2010. Ghana National Drugs Programme. Available at: http://ghndp.org
- Ghana National Health Insurance Scheme. Medicines List. 2011. Available at: http://www.nhis.gov.gh
- Ghana Ministry of Health. Ghana Standard Treatment Guidelines. Sixth edition. 2010. Ghana National Drugs Programme. Available at: http://ghndp.org
- Hien TT, Davis TME, Chuong LV, Sinh DXT, Phu NH, Agus C et al. Comparative Pharmacokinetics of Intramuscular Artesunate and Artemether in Patients with Severe Falciparum Malaria. Antimicrobial Agents and Chemotherapy. 2004; 48(11):4234-9
- Phu NH, Tuan PQ, Day N, Mai NTH, Chau TTH, Chuong LV et al. Randomized controlled trial of artesunate or artemether in Vietnamese adults with severe falciparum malaria. Malaria Journal. 2010;9:97
- McIntosh H, Olliaro P. Artemisinin derivatives for treating severe malaria. *Cochrane Database of Systematic Reviews* 2000,Issue 2. Art. No.: CD000527. DOI: 10.1002/14651858.CD000527.
- Matthew JL. Artemisinin Derivatives Versus Quinine for Severe Malaria in Children: A Systematic Review and Meta-Analysis. Indian Pediatrics. 2010; 47: 423-28
- Praygod G, de Frey A, Eisenhut M. Artemisinin derivatives versus quinine in treating severe malaria in children: a systematic review. Malaria Journal. 2008; 7:210
- Kyu HH, Fernandez E. Artemisinin derivatives versus quinine for cerebral malaria in African Children: a systematic review. Bulletin of the World Health Organization. 2009;87:896-904

Annex 1. Detailed search strategy and results

Set	Cochrane	PubMed	PubMed
1	arte*	arte* [Title/Abstract]	artesunate[Title/Abstract]
2	malaria	Severe malaria [Title/Abstract]	artemether[Title/Abstract]
3	1 AND 2	1 AND 2	Severe malaria [Title/Abstract]
4		limit 3 to reviews/meta-analyses/humans	1 AND 2 AND 3
5			Limit 4 to clinical trial/humans
**Search results**	**Cochrane**	**PubMed**	**PubMed**
Hits	16	59	7
Included	1	3	1
Excluded	15	56	6
Reason for exclusion			
Topic not relevant to this summary	14		6
Not a systematic review			
More complete reviews are available			
Additional reviews identified through reference lists			

Annex 2. Assessment of the risk of bias of a randomized controlled trial

Study reference: Phu NH, Tuan PQ, Day N, Mai NTH, Chau TTH, Chuong LV et al. Randomized controlled trial of artesunate or artemether in Vietnamese adults with severe falciparum malaria. Malaria Journal. 2010;9:97

Description of the study

**Study population:**	370 patients aged > 14 years admitted to the Hospital for Tropical Diseases in Ho Chi Minh city, Vietnam with severe malaria between 1996 and 2003
**Intervention:**	Intramuscular artesunate 2.4 mg/kg body weight on admission, then 1.2 mg/kg daily. Once oral therapy was tolerated oral artesunate was given to a total of 7 days.
**Control:**	Intramuscular artemether 3.2 mg/kg body weight on admission, followed by 1.6 mg/kg daily. Once oral therapy was tolerated oral artesunate was given to a total of 7 days.
**Outcomes:**	Mortality, time to parasite clearance, time to fever clearance, haematological recovery, time to coma recovery, adverse events including hypoglycaemia

Risk of Bias Criteria	Judgement
**1a. How did they generate a random sequence?**<br>*Examples of methods at low risk of selection bias: Computer randomisation, a random numbers table, shuffling cards, tossing a coin, drawing lots.*<br>'The randomization was generated from random number tables'	☐ High risk of bias<br>☐ Unclear risk<br>☑ Low risk of bias
**1b. How did they conceal allocation?**<br>*Examples of methods at low risk of selection bias: Centralized or telephone allocation, sequentially numbered, sealed, opaque envelopes.*<br>'Labels with the name of drug for each patient were put in coded sealed opaque envelopes…once a patient was enrolled in the study the envelope was opened'	☐ High risk of bias<br>☐ Unclear risk<br>☑ Low risk of bias
**3. Were patients and study staff blinded to which treatment the participant received?**<br>*Examples of outcomes at low risk of performance bias: If the outcome is objective (e.g. death) then blinding is less critical. If the outcome is subjective (e.g. symptoms or function) then blinding of the outcome assessor is critical.*<br>'An independent team of nurses not otherwise involved in the study or responsible for the care of these patients, opened the envelope, randomized the patient and prepared the injection. Neither the treating physicians, study doctors and nurses, or patients knew which anti-malarial drug was administered.'	☐ High risk of bias<br>☐ Unclear risk<br>☑ Low risk of bias
**4. Were outcome assessors blinded to which treatment the participant received?**<br>*Examples of outcomes at low risk of detection bias: If the outcome is objective (e.g. death) then blinding is less critical. If the outcome is subjective (e.g. symptoms or function) then blinding of the outcome assessor is critical.*<br>As above	☐ High risk of bias<br>☐ Unclear risk<br>☑ Low risk of bias
**5. Have missing data due to participant withdrawals from the study been handled appropriately?**<br>*Examples of low risk of attrition bias: Withdrawals are low (less than 10%) and the reasons for withdrawal are clearly stated and balanced between groups.*	☐ High risk of bias<br>☐ Unclear risk<br>☑ Low risk of bias
**6. Is there evidence of selective outcome reporting?**<br>*Examples of low risk studies: If the protocol is available and all pre-specified outcomes appear in the report, or if given the nature of the question all expected outcomes have been reported.*<br>The standard protocol for malaria trials is well established and all expected outcomes have been reported	☐ High risk of bias<br>☐ Unclear risk<br>☑ Low risk of bias
**7. Is there evidence of any other forms of bias?**<br>*Examples of other bias: One of the authors has a history of fraudulent reporting, stopping a trial early,*<br>This study was stopped early due to a declining incidence of severe malaria. This was done prior to unlocking of the study codes.	☐ High risk of bias<br>☐ Unclear risk<br>☑ Low risk of bias

For further information on the Cochrane Tool for Assessing the Risk of Bias see: Higgins JPT, Green S. Cochrane Handbook for Systematic Reviews of Interventions. Version 5.1.0 [updated March 2011]. The Cochrane Collaboration, 2011. Available at: www.cochrane-handbook.org

Annex 3. AMSTAR assessment of the systematic review

Review reference: Praygod G, de Frey A, Eisenhut M. Artemisinin derivatives versus quinine in treating severe malaria in children: a systematic review. Malaria Journal. 2008; 7:210

**1. Was an 'a priori' design provided?**<br>The research question and inclusion criteria should be established before the conduct of the review.	☐ Yes<br>☑ No<br>☐ Can't answer<br>☐ Not applicable
**2. Was there duplicate study selection and data extraction?**<br>There should be at least two independent data extractors and a consensus procedure for disagreements should be in place.	☐ Yes<br>☑ No<br>☐ Can't answer<br>☐ Not applicable
**3. Was a comprehensive literature search performed?**<br>At least two electronic sources should be searched. The report must include years and databases used (e.g. Central, EMBASE, and MEDLINE). Key words and/or MESH terms must be stated and where feasible the search strategy should be provided. All searches should be supplemented by consulting current contents, reviews, textbooks, specialized registers, or experts in the particular field of study, and by reviewing the references in the studies found.	☑ Yes<br>☐ No<br>☐ Can't answer<br>☐ Not applicable
**4. Was the status of publication (i.e. grey literature) used as an inclusion criterion?**<br>The authors should state that they searched for reports regardless of their publication type. The authors should state whether or not they excluded any reports (from the systematic review), based on their publication status, language etc.	☐ Yes<br>☑ No<br>☐ Can't answer<br>☐ Not applicable
**5. Was a list of studies (included and excluded) provided?**<br>A list of included and excluded studies should be provided.	☐ Yes<br>☑ No<br>☐ Can't answer<br>☐ Not applicable
**6. Were the characteristics of the included studies provided?**<br>In an aggregated form such as a table, data from the original studies should be provided on the participants, interventions and outcomes. The ranges of characteristics in all the studies analyzed e.g. age, race, sex, relevant socioeconomic data, disease status, duration, severity, or other diseases should be reported.	☑ Yes<br>☐ No<br>☐ Can't answer<br>☐ Not applicable
**7. Was the scientific quality of the included studies assessed and documented?**<br>'A priori' methods of assessment should be provided (e.g., for effectiveness studies if the author(s) chose to include only randomized, double-blind, placebo controlled studies, or allocation concealment as inclusion criteria); for other types of studies alternative items will be relevant.	☑ Yes<br>☐ No<br>☐ Can't answer<br>☐ Not applicable
**8. Was the scientific quality of the included studies used appropriately in formulating conclusions?**<br>The results of the methodological rigor and scientific quality should be considered in the analysis and the conclusions of the review, and explicitly stated in formulating recommendations.	☑ Yes<br>☐ No<br>☐ Can't answer<br>☐ Not applicable
**9. Were the methods used to combine the findings of studies appropriate?**<br>For the pooled results, a test should be done to ensure the studies were combinable, to assess their homogeneity (i.e. Chi-squared test for homogeneity, I^2). If heterogeneity exists a random effects model should be used and/or the clinical appropriateness of combining should be taken into consideration (i.e. is it sensible to combine?).	☐ Yes<br>☐ No<br>☑ Can't answer<br>☐ Not applicable
**10. Was the likelihood of publication bias assessed?**<br>An assessment of publication bias should include a combination of graphical aids (e.g., funnel plot, other available tests) and/or statistical tests (e.g., Egger regression test).	☐ Yes<br>☐ No<br>☑ Can't answer<br>☐ Not applicable
**11. Was the conflict of interest stated?**<br>Potential sources of support should be clearly acknowledged in both the systematic review and the included studies.	☑ Yes<br>☐ No<br>☐ Can't answer<br>☐ Not applicable

For further information on the AMSTAR tool see: Shea B, Grimshaw J, Wells G, Boers M, Andersson N, Hamel C, et al. Development of AMSTAR: a measurement tool to assess the methodological quality of systematic reviews. BMC Medical Research Methodology. 2007; 7(1):10.

Annex 4. Assessment of the local applicability of the evidence

References: Praygod 2008 and Phu 2010

1. Were the studies included in this systematic review conducted in settings similar to Ghana, or were the findings consistent across settings and time periods?

The trial of artesunate versus artemether was conducted in Adults in Asia, It is therefore poorly generalised to African children.

The trials included in Praygod 2008 were conducted in adults and children from a range of countries in Africa and Asia.

2. Are there important differences in on-the-ground realities and constraints in Ghana that might substantially alter the feasibility and acceptability of this drug/formulation?

No.

3. Are there important differences in health system arrangements that may mean this drug/formulation could not work in the same way?

No

4. Are there important differences in the baseline conditions that might yield different absolute effects even if the relative effectiveness was the same?

It is unclear. The data available from the Ghana NMCP suggest a very low mortality from severe malaria, which may be an artefact due to the way the data is collected. If true, the absolute benefit of a change to artesunate is likely to be lower..

5. What insights can be drawn about options, implementation, and monitoring and evaluation?

For further information on the SUPPORT tool used for this assessment see: Lavis JN, Oxman AD, Souza NM, Lewin S, Gruen RL, Fretheim A. SUPPORT Tools for evidence-informed health Policymaking (STP) 9: Assessing the applicability of the findings of a systematic review. Health Research Policy and Systems. 2009; 7 Suppl 1:S9
