# Supplementary material for: Integrating Global and National Knowledge to Select Medicines for Children: The Ghana National Drugs Programme
Source: PLoS Med. 2013 May 21;10(5):e1001449. doi: 10.1371/journal.pmed.1001449 (PMC3660441; doi:10.1371/journal.pmed.1001449)
Supplement: Text S3 — Zinc evidence summary. (PDF) [file pmed.1001449.s003.pdf]

Evidence Summary
For the Ghana Essential Medicines Committee

Title: # Zinc sulphate for treating diarrhoea in children

Formulation: Oral liquid 10mg/5ml; Dispersible tablet 10mg; Tablet (scored) 20 mg

Executive Summary

Context: The WHO recommends that children aged less than 5 years in low and middle income countries receive 14 days of zinc supplementation during and after episodes of diarrhoea.
Although also recommended in the Ghana STG, a paediatric formulation of zinc is not listed on the Ghana essential medicines list (EML).

Effects: **Benefits of zinc supplementation for diarrhoea**

- In children aged > 6 months zinc may decrease the duration of diarrhoea *(low quality evidence),*
- This effect is probably larger in children with persistent diarrhoea *(moderate quality evidence)*, or with signs of malnutrition *(high quality evidence)*.

Harms of zinc supplementation for diarrhoea

- In children aged < 6 months zinc may increase the duration of diarrhoea *(low quality evidence)*,
- Zinc increases vomiting compared to placebo in both age groups *(high quality evidence)*.

Feasibility: Dispersible tablets may have some programmatic advantages over oral solution

Acceptability: Dispersible tablets are already in use for some conditions.

Cost: $ 0.28 per patient

Conclusion: Zinc appears to be beneficial in children age > 6 months, especially those with signs of malnutrition

For consideration: Consider addition of oro-dispersible zinc tablets to the Ghana EML/NHIL for use in children aged > 6 months

About this evidence summary

Who prepared this summary? This summary was prepared by Saviour Yevutsey, Elizabeth Adjei-Acquah & Eugene Addo with technical support from the Liverpool School of Tropical Medicine.
Who funded this evidence summary? Training and technical support for the production of this summary was funded by the Bill and Melinda Gates Foundation through the 'Better Medicines for Children Project', co-ordinated by the World Health Organization, in partnership with the Ghana Ministry of Health.
Declaration of conflicts of interest: None declared

Context

Why should this drug/formulation be considered by the committee?

The management of acute diarrhoea in children requires effective rehydration with oral rehydration salts (ORS). Additional supplementation with 10 to 14 days of Zinc has been recommended by the World Health Organization (WHO) since 2004, for its reported benefits on the duration and severity of illness, and the prevention of further episodes (WHO 2004).

In Ghana, supplementation is recommended at 10mg per day for children aged < 6 months, and 20mg per day for those aged > 6 months (Ghana STG). However, at present the Ghana essential medicines list (EML) does not contain a suitable zinc formulation for administration to children (Ghana EML).

Zinc sulphate tablets, 10mg or 20mg, are reimbursable via the Ghana National Health Insurance Medicines list, but may not be palatable to small children (Ghana NHIL).

What questions does this evidence summary aim to address?

This evidence summary aims to answer the following questions:

1. **Does zinc supplementation reduce morbidity and mortality from acute diarrhoea in children under 5?**
2. **What is the likely public health impact of zinc supplementation for acute diarrhoea in Ghana?**
3. **Is there an acceptable and feasible formulation available for addition to the Ghana EML?**
4. **What are the resource implications of introducing this?**

Effects

Q1. Is oral Zinc superior to placebo for treating diarrhoea in children under 5?

How is diarrhoea treated and how might zinc supplementation work?

Acute diarrhoea remains a major cause of morbidity and mortality in low and middle income countries (Black 2010). It is usually of viral origin and the mainstay of management is effective rehydration using ORS. Antibiotics are usually not necessary but may be beneficial in specific infections (WHO 2004).

Zinc is an essential micronutrient found in more expensive foods such as meat and fish, and zinc deficiency is common among many populations due to inadequate dietary intake (IZINCG 2004). Infants are generally at lower risk of deficiency as they acquire adequate pre-term zinc stores regardless of maternal deficiency, and receive ongoing supplementation via breast-milk (Iqbal 2001, Krebs 1999). Zinc deficiency may be further exacerbated by bouts of diarrhoea due to excretion of zinc in the stools.

Zinc is known to play an important role in many cellular and immunological mechanisms. In the gastrointestinal system, it is involved in promoting mucosal integrity and local production of antibodies (Berni Canani 2010).

What research evidence is available?

In July, 2011, we searched the Cochrane Library and Pubmed for systematic reviews comparing oral zinc to placebo, for the treatment of acute diarrhoea.

We found one Cochrane review, up-to-date to November 2010 (Lazzerini 2010), and 3 recent non-Cochrane systematic reviews (Fischer Walker 2010, Haider 2009, Patro 2008). The most up-to date of these is the Cochrane review whose findings are summarized below. Older reviews were not examined.

What does the research show?

The Cochrane review aimed to summarize the benefits and harms of Zinc sulphate in both acute and persistent diarrhoea. Twenty-two randomized controlled trials were included, enrolling 8924 children.

The benefits of zinc supplementation in diarrhoea:

- ❖ There is not enough evidence from well conducted randomized controlled trials to be able to say whether zinc sulphate supplementation during acute diarrhoea reduces death or hospitalization.

In children older than 6 month with acute diarrhoea;

- ❖ Zinc sulphate may shorten the duration of diarrhoea by an average of 5.6 hours (95% CI 13.88 hours shorter to 2.63 hours longer, 2091 participants, 5 studies, *low quality evidence*),
- ❖ Zinc sulphate probably reduces the number of children whose diarrhoea persists until day 3 (RR 0.84, 95% CI 0.71 to 0.99, 1386 participants, 2 studies, *moderate quality evidence*).
- ❖ Zinc sulphate probably reduces the number of children whose diarrhoea persists until day 7 (RR 0.73, 95% CI 0.61 to 0.88, 3865 participants, 6 studies, *moderate quality evidence*).

In children older than 6 month with persistent diarrhoea;

- ❖ Zinc sulphate probably shortens the duration of diarrhoea by an average of 16.01 hours (95% CI 5.86 hours shorter to 26.16 hours shorter, 388 participants, 4 studies, *moderate quality evidence*),

In children older than 6 months with moderate malnutrition

- ❖ Zinc sulphate shortens the duration of diarrhoea by an average of 27 hours (95% CI 14.62 hours shorter to 39.34 hours shorter, 336 participants, 4 studies, *high quality evidence*).

About systematic reviews

What is a systematic review? A systematic review seeks to answer a well formulated and specific question by identifying, critically appraising, and summarising the results of all relevant trials, published and unpublished, according to pre-stated and transparent methods.
What is a Cochrane Systematic Review? The Cochrane Collaboration is an international network of more than 28,000 people from over 100 countries. The collaboration is one of the biggest producers of systematic reviews on the effects of healthcare interventions, and Cochrane Systematic Reviews are recognized internationally as the benchmark for high quality information. Over 4,600 reviews have now been published online in *The Cochrane Library*. http://www.thecochranelibrary.com
What about non-Cochrane systematic reviews? Non-Cochrane reviews can be variable in quality. Important predictors of quality are: a broad and exhaustive search strategy, an assessment of the risk of bias of included studies, and freedom from conflicts of interest.

The harms of zinc supplementation in diarrhoea:

In children younger than 6 months with acute diarrhoea;

- Zinc sulphate may increase the duration of diarrhoea by an average of 5.23 hours (95% CI 4 hours shorter to 14.45 hours longer, 1334 participants, 2 studies, *low quality evidence*),
- Zinc sulphate probably increases the number of children whose diarrhoea persists until day 7 (RR 1.24, 95% CI 0.99 to 1.54, 1074 participants, 1 study, *moderate quality evidence*)
- Zinc sulphate increases vomiting compared to placebo (RR 1.54, 95% CI 1.05 to 2.24, 1334 participants, 3 studies, *high quality evidence*).

In children older than 6 months;

- Zinc sulphate increases vomiting compared to placebo (RR1.56, 95% CI 1.32 to 1.85, 2340 participants, 5 studies, *high quality evidence*).

Agreements or disagreements with other reviews

These findings are consistent with the conclusions of Haider 2009 (which also notes the finding of no benefit in children aged less than 6 months), and Patro 2008.

The additional systematic review (Fischer Walker 2010) evaluated 13 trials and included two prominent effectiveness trials that were excluded from the Cochrane review (Baqui 2002 & Bhandari 2008). Based on the findings of these two trials the authors concluded that 'Zinc is an effective therapy for diarrhoea and will decrease diarrhoea mortality by 23%'.

In Bhandari 2008, health staff and caretakers in the intervention group received training in the management of acute diarrhoea, and the administration of both ORS and zinc. Consequently, the use of both ORS and zinc were significantly higher in the intervention group compared to control and the individual effect of zinc can not be determined.

It is less clear whether Bacqui 2002 should have been excluded from the Cochrane review. In this cluster-randomized trial, conducted in Bangladesh, the two groups seem to have been treated identically except for the use of zinc in the intervention group. However the use of ORS in the two groups is not reported. Overall mortality was not different between the two groups (33 in the intervention group vs 37 in the control group), but once deaths due to drowning were removed from the analysis the result appears statistically significant (13 in the intervention group vs 27 in the control group, Rate Ratio 0.49, 95% CI 0.25 to 0.94). The reason for the disparity in drowning deaths between the two groups is not clear but may reflect other underlying differences between the two groups.

Bacqui 2002 also reports statistically significant reductions in diarrhoea incidence and diarrhoea related hospitalizations in the clusters receiving zinc over a 2 year surveillance period.

A description of these two trials has been included in Annex 2.

About systematic reviews

What is a systematic review? A systematic review seeks to answer a well formulated and specific question by identifying, critically appraising, and summarising the results of all relevant trials, published and unpublished, according to pre-stated and transparent methods.
What is a Cochrane Systematic Review? The Cochrane Collaboration is an international network of more than 28,000 people from over 100 countries. The collaboration is one of the biggest producers of systematic reviews on the effects of healthcare interventions, and Cochrane Systematic Reviews are recognized internationally as the benchmark for high quality information. Over 4,600 reviews have now been published online in *The Cochrane Library*. http://www.thecochranelibrary.com
What about non-Cochrane systematic reviews? Non-Cochrane reviews can be variable in quality. Important predictors of quality are: a broad and exhaustive search strategy, an assessment of the risk of bias of included studies, and freedom from conflicts of interest.

Are the results of the research reliable?

How much confidence can we have in the systematic review methods?

The Cochrane review was well conducted, with only minor limitations (see Annex 3). The search strategy included the Cochrane Infectious Diseases Group Specialized Register, CENTRAL (The Cochrane Library 2010, Issue 11), MEDLINE, EMBASE, LILACS, CINAHL, mRCT, and reference lists.

The non-Cochrane systematic review (Fischer Walker 2010) had some major limitations in both the selection and assessment of the included trials, and the interpretation of the results (see Annex 3).

How much confidence can we have in the systematic review results?

The quality of the evidence provided by the Cochrane review has been assessed using GRADE. A summary of this assessment is shown in the Summary of Findings Table overleaf.

The quality of evidence provided by the review is variable with serious concerns about:

- the 'directness' of the evidence; with most of the trials being conducted in hospital settings in Asia, and
- the 'inconsistency' of results; with substantial heterogeneity between trials.

The observed heterogeneity between trial results suggests that the benefits of zinc may be dependant on important characteristics of the trials, the participants, or the interventions. The authors of the review conducted a sub-group analysis by age and nutritional status, and found more consistent benefits in the trials which included only children aged > 6 months or those including only children with signs of malnutrition.

Can the results of the research be applied to Ghana?

The International Zinc Nutrition Consultative group rates Ghana as a medium risk country for Zinc deficiency (iZiNCG 2004). This assessment was based on a nutritional evaluation of the typical diet, and the prevalence of potential surrogate markers such as stunting in pre-school children (height for age below -2SD), and anaemia.

The majority of the zinc supplementation studies have been conducted in countries at high risk of Zinc deficiency (India & Bangladesh) which may limit the applicability of this evidence. Only two trials are reported from Africa, from Ethiopia and Nigeria (both medium risk countries), and neither found a significant benefit with Zinc (Fischer Walker 2006, Fajolu 2008).

The Ghana DHS 2008, states that 28% of children under 5 are stunted (a marker for chronic undernutrition), with the highest levels in rural areas (32%), in the Eastern and Upper regions (38% and 36% respectively), and in children aged 18 to 23 months (40%). In addition 9% are wasted (weight for height below -2SD, a marker for recent undernutrition), with the highest rates among children age 6-8 months (29 percent), and in the Upper West (14 percent), Northern (13 percent) and Central (12 percent) regions.

About quality of evidence (GRADE)

The GRADE system considers 'quality' to be a judgment of the extent to which we can be confident that the estimates of effect are correct. The level of 'quality' is judged on a 4-point scale. Evidence from randomized controlled studies is initially graded as HIGH and downgraded by one, two or three levels after full consideration of : any limitations in the design of the studies, the directness (or applicability) of the evidence, and the consistency and precision of the results.

High: Further research is very unlikely to change our confidence in the estimate of effect.

Moderate: Further research is likely to have an important impact on our confidence in the estimate of effect and may change the estimate.

Low: Further research is very likely to have an important impact on our confidence in the estimate of effect and is likely to change the estimate

Very low: We are very uncertain about the estimate.

Summary of findings table 1: Children > 6 months

Zinc supplementation compared to placebo for treating diarrhoea

Patient or population: Children aged > 6 months
Settings: Low and middle income countries

Outcomes	Illustrative comparative risks* (95% CI)		Relative effect (95% CI)	No of Participants (studies)	Quality of the evidence (GRADE)
	Assumed risk<br>**Placebo**	Corresponding risk<br>**Zinc supplementation**			
**Death**	**3 per 1000**	**1 per 1000**<br>(0 to 9)	**RR 0.30**<br>(0.03 to 2.92)	1885<br>(4 studies)	**low**[1]
**Hospitalization**	**-**	**-**	**-**	891<br>(1 study)	**low**[2]
**Duration of diarrhoea**	**with acute diarrhoea**				
	The mean duration of diarrhoea ranged across control groups from **41 to 170 hours**	The mean duration of diarrhoea in the intervention groups was **5.62 hours shorter**<br>(13.88 shorter to 2.63 longer)		2091<br>(5 studies)	**low**[3]
	**with persistent diarrhoea**				
	The mean duration of diarrhoea ranged across control groups from **84 to 168 hours**	The mean duration of diarrhoea in the intervention groups was **15.84 hours shorter**<br>(25.43 shorter to 6.24 shorter)		529<br>(5 studies)	**moderate**[4]
	**with signs of moderate malnutrition**				
	The mean duration of diarrhoea ranged across control groups from **103 to 147 hours**	The mean duration of diarrhoea in the intervention groups was **26.98 hours shorter**<br>(39.34 shorter to 14.62 shorter)		336<br>(3 studies)	**high**[5,6]
**Diarrhoea on day 3**	**with acute diarrhoea**				
	**323 per 1000**	**271 per 1000**<br>(229 to 320)	**RR 0.84**<br>(0.71 to 0.99)	1386<br>(2 studies)	**moderate**[7]
**Diarrhoea on day 7**	**with acute diarrhoea**				
	**128 per 1000**	**93 per 1000**<br>(78 to 113)	**RR 0.73**<br>(0.61 to 0.88)	3865<br>(6 studies)	**moderate**[8]
**Adverse events (vomiting)**	**131 per 1000**	**204 per 1000**<br>(173 to 242)	**RR 1.56**<br>(1.32 to 1.85)	2340<br>(5 studies)	**high**[6]

*The illustrative **assumed risk** is taken from the data presented in the trials.
The **corresponding risk** (and its 95% confidence interval) is based on the assumed risk in the comparison group and the **relative effect** of the intervention (and its 95% CI).
CI: Confidence interval; **RR:** Risk ratio;

[1] In the four trials included in the Cochrane review which specifically stated whether deaths occurred, death was very rare. Downgraded under directness by two levels as three of these trials were conducted in hospitals and so may not accurately reflect the effect of this intervention when applied to communities.
[2] Hospitalizations were also very rare. Only one trial
[3] Five trials conducted in India (3), Bangladesh, and Nigeria presented data specifically for this age group. The quality of evidence was downgraded by 1 under directness as only one of these trials was conducted in Africa and this did not show an effect. Also downgraded under consistency due to moderate unexplained heterogeneity between trials. The result is not statistically significant.
[4] These 5 trials were conducted in Bangladesh (2), India, Peru and Pakistan. Downgraded by 1 under study limitations as 3 trials were at high or unclear risk of bias. There was no reason to downgrade under consistency, directness or precision.
[5] In a secondary subgroup analysis by nutritional status, the effect of zinc appears to be largest in malnourished children, and with no effect seen in the one trial which only included well nourished children.

These 3 trials were conducted in India, Bangladesh and Turkey. Although none of the trials were conducted in Africa, it seems likely that this effect is still present in malnourished children in Africa.
[6] There was no reason to downgrade under study limitations, consistency, directness or precision.
[7] These 2 studies were conducted in India and Peru. Downgrade by 1 for directness. This finding is consistent with the reduction in duration of diarrhoea, and in diarrhoea persisting to day 7
[8] In the 6 studies reporting this outcome there was a consistent reduction in the number of children with diarrhoea persisting until day 7. Downgraded by 1 under directness as none of these trials were conducted in Africa.

Summary of findings table 2: Children < 6 months

Zinc supplementation compared to placebo for treating diarrhoea

Patient or population: Children < 6 months
Settings: Low and middle income countries

Outcomes	Illustrative comparative risks* (95% CI)		Relative effect (95% CI)	No of Participants (studies)	Quality of the evidence (GRADE)
	Assumed risk<br>**Placebo**	Corresponding risk<br>**Zinc supplementation**			
**Death**	-	-	-	1334<br>(2 studies)	**low**[1,2]
**Hospitalization**	-	-	-	1074<br>(1 study)	**low**[3]
**Duration of diarrhoea**	The mean duration of diarrhoea ranged across control groups from<br>**98 to 133 hours**	The mean duration of diarrhoea in the intervention groups was **5.23 hours longer**<br>(4 shorter to 14.45 longer)		1334<br>(2 studies)	**low**[4]
**Diarrhoea on day 3**	-	-	-	(0 studies)	-
**Diarrhoea on day 7**	**203 per 1000**	**252 per 1000**<br>(201 to 313)	**RR 1.24**<br>(0.99 to 1.54)	1074<br>(1 study)	**moderate**[5]
**Adverse events (vomiting)**	**64 per 1000**	**99 per 1000**<br>(67 to 143)	**RR 1.54**<br>(1.05 to 2.24)	1334<br>(2 studies)	**high**[6]

*The illustrative **assumed risk** is taken from the data presented in the trials.
The **corresponding risk** (and its 95% confidence interval) is based on the assumed risk in the comparison group and the **relative effect** of the intervention (and its 95% CI).
CI: Confidence interval; **RR:** Risk ratio;

Footnotes
[1] Two studies have examined Zinc supplementation in children aged < 6 months. One was conducted in a hospital in Bangladesh and one in the community in Ethiopia, India and Pakistan.
[2] In these two studies deaths were very rare. There were no deaths in Bangladesh and one death in each group in the multicentre trial. Downgraded by two under precision as these trials are significantly underpowered to exclude an effect on mortality.
[3] Hospitalizations were also rare with only one being recorded in 1074 patients. Downgraded by 2 under precision.
[4] The quality of the evidence was downgraded by 1 under directness as only one of these trials was conducted in Africa, and also downgraded by 1 under precision as the result is not statistically significant.
[5] This multi-centre study from India, Pakistan and Ethiopia found that Zinc increased the number of infants with diarrhoea persisting until day 7. The result was consistent across the trial settings and approaches statistical significance. Downgraded by 1 under directness as it is not yet possible to generalise this to all settings.
[6] There was no reason to downgrade under study limitations, consistency, directness or precision.

Q2. What is the potential public health impact of applying these results to Ghana?

Diarrhoea is a major cause of morbidity in children aged less than 5-years in Ghana, accounting for approximately 5% of hospital admissions, and 3% of deaths (Facts and Figures, 2009).

The data from well conducted randomized controlled trials has not demonstrated a statistically significant benefit on mortality or hospitalisation.

The public health benefit of a reduction in diarrhoea duration is not clear.

Q3. Is there an acceptable and feasible formulation suitable for use in Ghana?

Description of the formulations

	Oral solution 10mg/5ml	Oro-dispersible tablet
**Route of administration:**	Oral	Oral
**Additional requirements:**	Spoon	Dissolve in water
**Storage:**	?	Store at room temperature
**Stability:**	?	?
**Transport:**	Bulky	Less bulky

Is the introduction of this formulation feasible?

**Locally available manufacturers:**	M&G Pharmaceuticals Ltd
**Ghana FDB Registration:**	Yes
**International manufacturers:**	10mg/5ml solution: Incepta, Bangladesh & Zenufa,Tanzania<br>10mg dispersible tablet: Banner, Canada & Esco/Macleods, India
**Suggested level of prescribing:**	All levels
**Educational requirements:**	Ghana DS 2008 suggests less than 2% of diarrhoea episodes are treated with Zinc. Widespread roll out would require extensive education (DHS 2008).
**System requirements:**	None
**Any other concerns:**	None

Will the introduction of this formulation be acceptable to all stakeholders?

**Toxicity:**	
**Appropriateness of formulation:**	Both formulations are appropriate for this age group, however dispersible tablets may have some programmatic advantages
**Additional Stakeholders:**	National Health Insurance Authority, prescribers
**National Guidelines:**	Zinc is already recommended in the Ghana STG
**International Guidelines**	The Ghana STG recommendations are in line with current WHO guidance.

Cost

Q. What are the resource implications?

What do the different formulations cost?

	Zinc Sulphate formulation	Median	Minimum	Maximum
**IDPI Price Guide:**	No pediatric formulation listed	-	-	-
**WHO Sources and prices 2nd edition:**	Oral liquid 10mg/5ml	$ 0.021 per 5ml	$ 0.021	$ 0.021
	Dispersible tablet 10mg	$ 0.010 per tab	$ 0.008	$ 0.019
	Tablet (scored) 20mg	$ 0.020 per tab	$ 0.011	$ 0.060

The median cost of adding 14 days of zinc supplementation to normal care in children aged over 6 months would be $0.28, or 0.44 Ghana Cedis, per patient (orodispersible tablet).

Is it cost-effective?

We searched the database of economic evaluations within the Cochrane library for economic evaluations of zinc supplementation. We found one study from the Philippines (Gregorio 2007). The methodological quality of this economic evaluation was assessed using a check list given in Annex 5.

This study randomized 60 children aged 2 to 59 months with acute diarrhoea to management with ORS alone or ORS plus 14 days of Zinc supplementation. The primary outcome for effectiveness was a reduction in the mean duration of diarrhoea of 17 hours.

A societal perspective was taken for the cost analysis which sought to measure the direct medical cost, the out-of-pocket expenditure to the patients family (travel, food etc), and the indirect cost to the caretaker or the child through loss of time/work.

The only costs which differed significantly between treatment groups were the cost of drugs and medical supplies, and the indirect cost to the child caretaker.

The addition of zinc supplementation appeared to treble the cost of drug treatment in acute diarrhoea.

The authors of the paper argue that this increase in cost is balanced by a saving to society of the reduction in caretakers time devoted to caring for the child. It is arguable that this represents a genuine societal saving where the caretaker is a full-time mother.

The cost-effectiveness of zinc in the treatment of acute diarrhoea therefore remains unclear.

About the NHS Economic Evaluations Database within the Cochrane Library

As healthcare resources are finite, information about both costs and effects are essential to making evidence-based decisions about competing healthcare interventions. But information about cost-effectiveness can be difficult to identify, appraise and interpret.
The NHS Economic Evaluation Database (EED) assists decision-makers by systematically identifying economic evaluations from around the world, appraising their quality, and highlighting their relative strengths and weaknesses.
The NHS Economic Evaluations Database is produced by the Centre for Reviews and Dissemination (CRD) at the University of York, UK.

References

- World Health Organization. Dept. of Child and Adolescent Health and Development/UNICEF. Clinical management of acute diarrhoea: WHO/UNICEF joint statement [WHO/FCH/CAH/04.7; UNICEF/PD/Diarrhoea/01]. Geneva: World Health Organization, 2004.
- Ghana Ministry of Health. Ghana Standard Treatment Guidelines. Sixth edition. 2010. Ghana National Drugs Programme. Available at: http://ghndp.org
- Ghana Ministry of Health. Ghana Essential Medicines List, Sixth edition. 2010. Ghana National Drugs Programme. Available at: http://ghndp.org
- Black RE, Cousens S, Johnson HL, Lawn JE, Rudan J, Bassani DB, et al. Global, regional, and national causes of child mortality in2008: a systematic analysis. Lancet 2010;375(9730):1969-87.
- International Zinc Nutrition Consultative Group (IZiNCG); Hotz C, Brown KH, editors. Assessment of the risk of zinc deficiency in population and options for its control [Technical Document]. Food and Nutrition Bulletin 2004;25(1 Suppl 2):94-204.
- Iqbal AS, Shahidullah M, Islam MN, Akhter S, Banu S. Serum zinc and copper levels in the maternal blood and cord blood of neonates. Indian Journal of Pediatrics 2001;68(6):523-6.
- Krebs NF. Zinc transfer to the breastfed infant. Journal of Mammary Gland Biology and Neoplasia 1999;4(3):259-68.
- Berni Canani R, Buccigrossi V, Passariello A. Mechanisms of action of zinc in acute diarrhea. Current Opinion Gastroenterology 2011;1:8-12.
- Lazzerini M, Ronfani L. Oral zinc for treating diarrhoea in children. *Cochrane Database of Systematic Reviews* 2008, Issue 3. Art. No.: CD005436. DOI: 10.1002/14651858.CD005436.pub2.
- Fischer Walker CL, Black RE. Zinc for the treatment of diarrhoea: effect on diarrhoea morbidity, mortality and incidence of future episodes. International Journal of Epidemiology 2010;39:i63–i69
- Haider BA, Bhutta ZA. The effect of therapeutic zinc supplementation among young children with selected infections:a review of the evidence. Food and Nutrition Bulletin 2009;30:S41-59.
- Patro B, Golicki D, Szajewska H. Meta-analysis: zinc supplementation for acute gastroenteritis in children. Alimentary Pharmacology & Therapeutics. 2008 Sep 15;28(6):713-23.
- Baqui AH, Black RE, El Arifeen S, Yunus M, Chakraborty J, Ahmed S, et al. Effect of zinc supplementation started during diarrhoea on morbidity and mortality in Bangladeshi children: community randomised trial. BMJ 2002;325(7372):1059-64.
- Bhandari N, Mazumder S, Taneja S, Dube B, Agarwal RC, Mahalanabis D, et al. Effectiveness of zinc supplementation plus oral rehydration salts compared with oral rehydration salts alone as a treatment for acute diarrhea in a primary care setting: a cluster randomized trial. Pediatrics 2008;121(5):e1279-85.
- Ghana Health Service. Facts and Figures. 2009. Available at: http://www.ghanahealthservice.org/includes/upload/publications/Facts%20and%20Figures%202009.pdf Accessed 20 October 2011.

Annex 1. Detailed search strategy and results

Set		Cochrane	PubMed
1		zinc	zinc [Title/Abstract]
2		diarrhoea	diarrhoea [Title/Abstract]
3		1 AND 2	1 AND 2
4			limit 3 to reviews or meta-analyses
**Search results**		**Cochrane**	**PubMed**
Hits		6	36
Included		1	3
Excluded		5	33
Reason for exclusion	Topic not relevant to this summary	5	
	Not a systematic review	-	
	More complete reviews are available	-	
Additional reviews identified through reference lists		1	

Annex 2. Characteristics of community effectiveness studies

Baqui 2002

**Methods**	**Trial design**: Cluster randomized trial<br>**Unit of randomization**: 'service areas'<br>**Intervention group**:15 clusters, containing 2483 eligible children at start of trial<br>**Control Group**: 15 clusters, containing 2502 eligible children at start of trial<br>**Data collection**: Bimonthly home visits from data collectors to collect data on diarrhoea morbidity and adherence to zinc. Recall data was collected for diarrhoea in the past week, and pneumonia in the past 2 weeks. Data on hospitalisations and death were collected via the routine health surveillance.
**Study context**	**Year of study:** Nov 1998 to Oct 2000<br>**Country:** Bangladesh<br>**Setting:** Rural (Matlab has been used as a field research site for the International centre for Diarrhoeal Disease Research since 1960). A health and surveillance demographic system now operates in 144 villages with 220,000 people.<br>**Health services available**: A diarrhoea treatment centre, community health workers (1 per 2000 population) and 'Bari' mothers (community volunteers , 1 per 50 population)<br>**Target group**: Children aged 3 to 59 months
**Intervention**	CHWs and 'Bari' mothers taught mothers in the intervention clusters to administer zinc at 20mg/day for 14 days regardless of the duration of the episode.
**Control**	Children in both the intervention and control clusters received ORS. All mothers were given advice on feeding and referral.
**Outcomes**	**Duration of diarrhoea:** Mean duration 4.6 days intervention group vs 5.8 days control group.<br>**Incidence of diarrhoea:** Rate ratio; 0.85, 95% CI 0.76 to 0.96, absolute reduction in incidence rate; -2.9% (-0.8% to -5.1%)<br>**Incidence of pneumonia:** Rate ratio; 0.93, 95% CI 0.78 to 1.10, absolute reduction in incidence rate; -0.7% (-2.0% to +0.7)<br>**Hospitalisation for diarrhoea:** Rate ratio; 0.76, 95% CI 0.59 to 0.98, absolute reduction in hospitalisation rate; - 2.2%, 95% CI -4.50 % to +0.04%<br>**Hospitalisation for pneumonia:** Rate ratio; 0.81, 95% CI 0.53 to 1.23, absolute reduction in hospitalisation rate; -0.9%, 95% CI -3.6% to +1.8%.<br>**Non-injury related deaths:** Rate ratio; 0.49, 95% CI 0.25 to 0.94, absolute reduction in death rate; -2.2%, 95% CI -3.7% to -0.6%.
**Other**	Study sponsor: Johns Hopkins Family Health and Child Survival Cooperative Agreement and ICDDR,B Cooperative Agreement, with funding from the US Agency for International Development.

Bhandara 2008

**Methods**	**Trial design:** Cluster randomized trial<br>**Unit of randomization:** A primary healthcare centre<br>**Intervention group:** 3 primary healthcare centres<br>**Control Group:** 3 primary healthcare centres<br>**Data collection:** Two cross-sectional surveys of households with children aged between 1 and 4 years, conducted at one and six months after the intervention began
**Study context**	**Year of study:** Jan 2005 to Sept 2006<br>**Country:** India<br>**Setting:** Six primary healthcare centres

	**Health services available:** Governmental workers at PHCs, private practitioners (without medical degrees), and community workers (1 per 1000)<br>**Target group:** Children aged 1 month to 4 years
**Intervention**	All health workers, including government, private and community workers, underwent refresher training in the management of diarrhoea, appropriate use of zinc, logistics and supplies, and referral criteria (½ day for physicians, 1 day for others).
**Control**	All health workers underwent the same training as the intervention group except the use of zinc was not discussed.
**Outcomes**	**ORS use was significantly different in the two groups:** 31.7 % intervention group versus 6.3% control group at 1 month. This is a significant confounder and so the effect of zinc can not be determined in this trial.
**Other**	**Study sponsor:** United Nations Children's Fund (New Delhi) and Department of biotechnology, Government of India (New Delhi). Department of Child and Adolescent Health and Development, World Health Organization (Geneva) provided the zinc tablets. US Agency for International Development provided financial support for the pilot project.

Annex 3. AMSTAR assessment of the Cochrane review (Lazzerini 2010)

Review reference: Lazzerini M, Ronfani L. Oral zinc for treating diarrhoea in children. *Cochrane Database of Systematic Reviews* 2008, Issue 3. Art. No.: CD005436. DOI: 10.1002/14651858.CD005436.pub2.

**1. Was an 'a priori' design provided?**<br>The research question and inclusion criteria should be established before the conduct of the review.	☑ Yes<br>☐ No<br>☐ Can't answer<br>☐ Not applicable
**2. Was there duplicate study selection and data extraction?**<br>There should be at least two independent data extractors and a consensus procedure for disagreements should be in place.	☑ Yes<br>☐ No<br>☐ Can't answer<br>☐ Not applicable
**3. Was a comprehensive literature search performed?**<br>At least two electronic sources should be searched. The report must include years and databases used (e.g. Central, EMBASE, and MEDLINE). Key words and/or MESH terms must be stated and where feasible the search strategy should be provided. All searches should be supplemented by consulting current contents, reviews, textbooks, specialized registers, or experts in the particular field of study, and by reviewing the references in the studies found.	☑ Yes<br>☐ No<br>☐ Can't answer<br>☐ Not applicable
**4. Was the status of publication (i.e. grey literature) used as an inclusion criterion?**<br>The authors should state that they searched for reports regardless of their publication type. The authors should state whether or not they excluded any reports (from the systematic review), based on their publication status, language etc.	☐ Yes<br>☑ No<br>☐ Can't answer<br>☐ Not applicable
**5. Was a list of studies (included and excluded) provided?**<br>A list of included and excluded studies should be provided.	☑Yes<br>☐ No<br>☐ Can't answer<br>☐ Not applicable
**6. Were the characteristics of the included studies provided?**<br>In an aggregated form such as a table, data from the original studies should be provided on the participants, interventions and outcomes. The ranges of characteristics in all the studies analyzed e.g. age, race, sex, relevant socioeconomic data, disease status, duration, severity, or other diseases should be reported.	☑ Yes<br>☐ No<br>☐ Can't answer<br>☐ Not applicable
**7. Was the scientific quality of the included studies assessed and documented?**<br>'A priori' methods of assessment should be provided (e.g., for effectiveness studies if the author(s) chose to include only randomized, double-blind, placebo controlled studies, or allocation concealment as inclusion criteria); for other types of studies alternative items will be relevant.	☑ Yes<br>☐ No<br>☐ Can't answer<br>☐ Not applicable
**8. Was the scientific quality of the included studies used appropriately in formulating conclusions?**<br>The results of the methodological rigor and scientific quality should be considered in the analysis and the conclusions of the review, and explicitly stated in formulating recommendations.	☑ Yes<br>☐ No<br>☐ Can't answer<br>☐ Not applicable
**9. Were the methods used to combine the findings of studies appropriate?**<br>For the pooled results, a test should be done to ensure the studies were combinable, to assess their homogeneity (i.e. Chi-squared test for homogeneity, I^2). If heterogeneity exists a random effects model should be used and/or the clinical appropriateness of combining should be taken into consideration (i.e. is it sensible to combine?).	☑ Yes<br>☐ No<br>☐ Can't answer<br>☐ Not applicable
**10. Was the likelihood of publication bias assessed?**<br>An assessment of publication bias should include a combination of graphical aids (e.g., funnel plot, other available tests) and/or statistical tests (e.g., Egger regression test).	☐ Yes<br>☑ No<br>☐ Can't answer<br>☐ Not applicable
**11. Was the conflict of interest stated?**<br>Potential sources of support should be clearly acknowledged in both the systematic review and the included studies.	☑ Yes<br>☐ No<br>☐ Can't answer<br>☐ Not applicable

For further information on the AMSTAR tool see: Shea B, Grimshaw J, Wells G, Boers M, Andersson N, Hamel C, et al. Development of AMSTAR: a measurement tool to assess the methodological quality of systematic reviews. BMC Medical Research Methodology. 2007; 7(1):10.

AMSTAR assessment of Fischer Walker 2010

Review reference: Fischer Walker CL, Black RE. Zinc for the treatment of diarrhoea: effect on diarrhoea morbidity, mortality and incidence of future episodes. International Journal of Epidemiology 2010;39:i63–i69

1. Was an 'a priori' design provided?
The research question and inclusion criteria should be established before the conduct of the review.

- ☐ Yes
- ☐ No
- ☑ Can't answer
- ☐ Not applicable

2. Was there duplicate study selection and data extraction?
There should be at least two independent data extractors and a consensus procedure for disagreements should be in place.

- ☐ Yes
- ☐ No
- ☑ Can't answer
- ☐ Not applicable

3. Was a comprehensive literature search performed?
At least two electronic sources should be searched. The report must include years and databases used (e.g. Central, EMBASE, and MEDLINE). Key words and/or MESH terms must be stated and where feasible the search strategy should be provided. All searches should be supplemented by consulting current contents, reviews, textbooks, specialized registers, or experts in the particular field of study, and by reviewing the references in the studies found.

- ☐ Yes
- ☐ No
- ☑ Can't answer
- ☐ Not applicable

4. Was the status of publication (i.e. grey literature) used as an inclusion criterion?
The authors should state that they searched for reports regardless of their publication type. The authors should state whether or not they excluded any reports (from the systematic review), based on their publication status, language etc.

- ☐ Yes
- ☑ No
- ☐ Can't answer
- ☐ Not applicable

5. Was a list of studies (included and excluded) provided?
A list of included and excluded studies should be provided.

- ☐ Yes
- ☑ No
- ☐ Can't answer
- ☐ Not applicable

6. Were the characteristics of the included studies provided?
In an aggregated form such as a table, data from the original studies should be provided on the participants, interventions and outcomes. The ranges of characteristics in all the studies analyzed e.g. age, race, sex, relevant socioeconomic data, disease status, duration, severity, or other diseases should be reported.

- ☐ Yes
- ☑ No
- ☐ Can't answer
- ☐ Not applicable

7. Was the scientific quality of the included studies assessed and documented?
'A priori' methods of assessment should be provided (e.g., for effectiveness studies if the author(s) chose to include only randomized, double-blind, placebo controlled studies, or allocation concealment as inclusion criteria); for other types of studies alternative items will be relevant.

- ☑ Yes
- ☐ No
- ☐ Can't answer
- ☐ Not applicable

8. Was the scientific quality of the included studies used appropriately in formulating conclusions?
The results of the methodological rigor and scientific quality should be considered in the analysis and the conclusions of the review, and explicitly stated in formulating recommendations.

- ☐ Yes
- ☑ No
- ☐ Can't answer
- ☐ Not applicable

9. Were the methods used to combine the findings of studies appropriate?
For the pooled results, a test should be done to ensure the studies were combinable, to assess their homogeneity (i.e. Chi-squared test for homogeneity, I^2). If heterogeneity exists a random effects model should be used and/or the clinical appropriateness of combining should be taken into consideration (i.e. is it sensible to combine?).

- ☐ Yes
- ☑ No
- ☐ Can't answer
- ☐ Not applicable

10. Was the likelihood of publication bias assessed?
An assessment of publication bias should include a combination of graphical aids (e.g., funnel plot, other available tests) and/or statistical tests (e.g., Egger regression test).

- ☐ Yes
- ☑ No
- ☐ Can't answer
- ☐ Not applicable

11. Was the conflict of interest stated?
Potential sources of support should be clearly acknowledged in both the systematic review and the included studies.

- ☑ Yes
- ☐ No
- ☐ Can't answer
- ☐ Not applicable

For further information on the AMSTAR tool see: Shea B, Grimshaw J, Wells G, Boers M, Andersson N, Hamel C, et al. Development of AMSTAR: a measurement tool to assess the methodological quality of systematic reviews. BMC Medical Research Methodology. 2007; 7(1):10.

Annex 4. Assessment of the local applicability of the systematic review (SUPPORT tool 9)

Review reference: Lazzerini M, Ronfani L. Oral zinc for treating diarrhoea in children. *Cochrane Database of Systematic Reviews* 2008, Issue 3. Art. No.: CD005436. DOI: 10.1002/14651858.CD005436.pub2.

1. Were the studies included in this systematic review conducted in settings similar to Ghana, or were the findings consistent across settings and time periods?

No, the majority of studies were conducted in hospitals in Asia. It is therefore difficult to generalise to a community level in Africa

No, there was substantial heterogeneity between trials with inconsistency related to age of participants, and level of malnutrition. There may also be other important factors influencing the heterogeneity such as zinc status, zinc dose, cause of diarrhoea

2. Are there important differences in on-the-ground realities and constraints in Ghana that might substantially alter the feasibility and acceptability of this drug/formulation?

No

3. Are there important differences in health system arrangements that may mean this drug/formulation could not work in the same way?

The studies were mainly in hospital settings. Compliance with the 14 day course may be compromised in a community setting due to vomiting or the taste of the product.

4. Are there important differences in the baseline conditions that might yield different absolute effects even if the relative effectiveness was the same?

Yes, the applicability of the results to Ghana will depend on the zinc and nutritional status of children in Ghana.

In order to assess this we will need to search for evidence from nutritional surveys in Ghana

5. What insights can be drawn about options, implementation, and monitoring and evaluation?

Unclear

For further information on the SUPPORT tool used for this assessment see: Lavis JN, Oxman AD, Souza NM, Lewin S, Gruen RL, Fretheim A. SUPPORT Tools for evidence-informed health Policymaking (STP) 9: Assessing the applicability of the findings of a systematic review. Health Res Policy Syst 2009, 7 Suppl 1:S9

Annex 5. Assessment of the economic evaluation

Cost effectiveness reference:		Comment
1. Is the study population clearly described?	☑ Yes ☐ No ☐ Unclear	Children 2-59m with acute diarrhoea
2. Are competing alternatives clearly described?	☑ Yes ☐ No ☐ Unclear	ORS+Zn vs ORS
3. Is a well-defined research question posed in answerable form?	☑ Yes ☐ No ☐ Unclear	
4. Is the economic study design appropriate to the stated objective?	☐ Yes ☐ No ☐ Unclear	
5. Is the chosen time horizon appropriate to include relevant costs and consequences?	☑ Yes ☐ No ☐ Unclear	
6. Is the actual perspective chosen appropriate?	☐ Yes ☑ No ☐ Unclear	Societal is probably inappropriate in this case
7. Are all important and relevant costs for each alternative identified?	☑ Yes ☐ No ☐ Unclear	
8. Are all costs measured appropriately in physical units?	☑ Yes ☐ No ☐ Unclear	
9. Are costs valued appropriately?	☐ Yes ☑ No ☐ Unclear	The societal gain to caretakers time may be misleading
10. Are all important and relevant outcomes for each alternative identified?	☑ Yes ☐ No ☐ Unclear	
11. Are all outcomes measured appropriately?	☑ Yes ☐ No ☐ Unclear	
12. Are outcomes valued appropriately?	☐ Yes ☐ No ☐ Unclear	
13. Is an incremental analysis of costs and outcomes of alternatives performed?	☑ Yes ☐ No ☐ Unclear	
14. Are all future costs and outcomes discounted appropriately?	☐ Yes ☐ No ☐ Unclear	Not applicable
15. Are all important variables, whose values are uncertain, appropriately subjected to sensitivity analysis?	☐ Yes ☐ No ☐ Unclear	
16. Do the conclusions follow from the data reported?	☐ Yes ☐ No ☐ Unclear	
17. Does the study discuss the generalizability of the results to other settings and patient/client groups?	☐ Yes ☐ No ☐ Unclear	
18. Does the article indicate that there is no potential conflict of interest of study researcher(s) and funder(s)?	☑ Yes ☐ No ☐ Unclear	
19. Are ethical and distributional issues discussed appropriately?	☐ Yes ☐ No ☑ Unclear	

For further information on the CHEC-list used for this assessment see: Evers S, Goossens M, de Vet H, van Tulder M, Ament A: Criteria list for assessment of methodological quality of economic evaluations: Consensus on Health Economic Criteria. International Journal of Technology Assessment in Health Care. 2005; 21:240-5.
