# Supplementary material for: Integrating Global and National Knowledge to Select Medicines for Children: The Ghana National Drugs Programme
Source: PLoS Med. 2013 May 21;10(5):e1001449. doi: 10.1371/journal.pmed.1001449 (PMC3660441; doi:10.1371/journal.pmed.1001449)
Supplement: Text S4 — Chlorhexidine evidence summary. (PDF) [file pmed.1001449.s004.pdf]

Evidence Summary
For the Ghana Essential Medicines Committee

Title: **Chlorhexidine for preventing neonatal cord infection**

Formulation: Chlorhexidine solution 5%

Executive Summary

Context: Infection causes one third of all neonatal deaths, and the healing umbilical stump may be the source of up to 50% of these infections.
Until recently there has been no reliable evidence to support any practice other than keeping the cord clean and dry. However, the results of one large community study of chlorhexidine cord care in Nepal are now available and 3 further studies, 2 in Africa, are underway.

Effects: **Benefits of chlorhexidine in neonatal cord care**

- Chlorhexidine may reduce neonatal mortality compared to soap and water or dry cord care *(low quality evidence)*,
- To be effective cleansing probably needs to begin on the first day of life *(moderate quality evidence)*.
- Chlorhexidine probably reduces severe and moderate cord infections compared to soap and water or dry cord care *(moderate quality evidence)*.

Harms of chlorhexidine in neonatal cord care

- Contact dermatitis has been reported with chlorhexidine but none were reported in this trial after application to over 5,000 neonates.

Feasibility: To achieve this effect mothers were visited at home a median of 6 times during the first 12 days.

Acceptability: No concerns

Cost: No economic evaluations were found.

Conclusion: Effectiveness studies of chlorhexidine use by mothers without additional post-natal visits would be useful.

For consideration: Consider addition to Ghana EML/NHIL
Consider national guidance on chlorhexidine use.

About this evidence summary

Who prepared this summary? This summary was prepared by Irene Andoh, Gertrude Dorcus Laryea & Taiiba Jibril Afaa with technical support from the Liverpool School of Tropical Medicine.

Who funded this evidence summary? This evidence summary was funded by the Bill and Melinda Gates Foundation through the 'Better Medicines for Children Project'; co-ordinated by the World Health Organization, in partnership with the Ghana Ministry of Health National Drugs Programme.
Declaration of conflicts of interest: None declared

Context

Why should this drug/formulation be considered by the committee?

The World Health Organisation recommendations on neonatal cord care have not changed since 1998 (WHO 1998). At that time there was no reliable evidence to support any practice other than keeping the cord clean and dry (Zupan 2004).

Two chlorhexidine formulations have now been added to the WHO model essential medicines list for children, for use in cord care where the risk of infection is high (WHO EML); 5% Chlorhexidine digluconate, and 20% chlorhexidine digluconate for dilution prior to use.

The Ghana EML currently lists; 1% Cream, 2.5% Solution, and 4% in a detergent base (Ghana EML). In the absence of any national guidance current practice is variable with alcohol, povidine iodine and traditional medicines often applied by health staff or mothers.

What questions does this evidence summary aim to address?

This evidence summary aims to answer the following questions:

1. **Does chlorhexidine cord cleansing reduce neonatal mortality and cord infections?**
2. **What is the potential public health impact of this intervention?**
3. **Is this formulation suitable for use in Ghana?**
4. **What are the resource implications of this change?**

Effects

Q1. Does chlorhexidine reduce neonatal mortality and cord infections?

What causes neonatal cord infection and how might chlorhexidine work?

Of the 4 million neonatal deaths which occur each year worldwide, almost all (99%) occur in low and middle countries, and up to a third are due to infection (Lawn 2005). The umbilical stump is a major entry point for invasive pathogens and cord infection may be the initiating event in almost 50% of hospital admissions for neonatal sepsis (WHO 1998).

Chlorhexidine is a bisbiguanide compound that acts by binding to the bacterial cell wall and disrupting its membrane, leading to increased permeability and cell content leakage (Russell 1986). It has a broad-spectrum of activity against both gram-positive and gram-negative organisms.

Systemic absorption of chlorhexidine through the skin has been reported in neonates. A review of safety concluded that despite widespread use, and published trials involving tens of thousands of neonates, reports of adverse events are rare (Mullany 2006a)

What research evidence is available?

In August 2011, we searched the Cochrane library and PubMed for systemic reviews comparing chlorhexidine solution with standard treatments for neonatal cord care. The search strategy is detailed in Annex 1.

We found one Cochrane review, up-to-date to 2004, and two more-recent non-Cochrane reviews relevant to this question (Blencowe 2011, Mcrury 2007). Each of these reviews only included one trial using chlorhexidine for neonatal cord care (Mullany 2006b).

What does the research show?

The Cochrane review included 21 studies comparing various antiseptics and antibiotics, mainly from hospital settings in developed countries. Despite including almost 9,000 neonates no systemic infections or deaths were observed.

The Nepalese trial (Mullany 2006b) was a cluster randomized trial enrolling 15123 neonates. Randomization was to cord cleansing with either chlorhexidine 4% solution or soap and water, or to dry cord care.

The benefits of using chlorhexidine:

- Chlorhexidine cord care may reduce neonatal mortality compared to dry cord care (RR 0.78, 95% CI 0.57 to 1.07, 10016 participants, 1 trial, *low quality evidence*), and soap and water (RR 0.78, 95% CI 0.57 to 1.07, 10032 participants, 1 trial, *low quality evidence*).
- To be effective cleansing probably needs to begin within the first 24 hours of delivery (*moderate quality evidence*), and may be ineffective after this (*low quality evidence*).
- Chlorhexidine probably reduces the risk of moderate to severe superficial cord infections with the size of the effect varying from a 75% reduction to a 32% reduction dependant on the definition used (15123 participants, 1 trial, *moderate quality evidence*).

The harms of using chlorhexidine:

- No adverse events were reported in this trial.

This is currently the only published trial designed to assess the impact of chlorhexidine on neonatal mortality. However, further large community trials are underway in Bangladesh, Zambia and Tanzania. The Bangladesh trial was due to report in 2010 but remains unpublished (Mullany 2009). The African trials are due in 2013 (ZamCAT).

About systematic reviews

What is a systematic review? A systematic review seeks to answer a well formulated and specific question by identifying, critically appraising, and summarising the results of all relevant trials, published and unpublished, according to pre-stated and transparent methods.

What is a Cochrane Systematic Review? The Cochrane Collaboration is an international network of more than 28,000 people from over 100 countries. The collaboration is one of the biggest producers of systematic reviews on the effects of healthcare interventions, and Cochrane Systematic Reviews are recognized internationally as the benchmark for high quality information. Over 4,600 reviews have now been published online in *The Cochrane Library*. http://www.thecochranelibrary.com

What about non-Cochrane systematic reviews? Non-Cochrane reviews can be variable in quality. Important predictors of quality are: a broad and exhaustive search strategy, an assessment of the risk of bias of included studies, and freedom from conflicts of interest.

Are the results of the research reliable?

How much confidence can we have in the trial methods?

The Nepalese trial was assessed using the Cochrane tool for assessing the risk of bias (see annex 2). The researchers adequately concealed treatment allocation to reduce the risk of selection bias. Although attempts were made to blind the outcome assessors to treatment, true blinding seems unlikely given the nature of the interventions. Subjective outcomes such as grading of umbilical stump redness may therefore be at high risk of bias.

How much confidence can we have in the trial results?

The quality of the evidence provided by this trial has been assessed using the methods developed by the GRADE working group. A summary of the main results of the review, and the quality assessments is shown overleaf in the Summary of Findings table.

The evidence for a reduction in mortality was downgraded due to concerns about:

- the 'directness' of the evidence; as only one trial has been conducted in a very specific population, and
- the 'precision' of the estimate; although the risk of death was lower with chlorhexidine, this did not reach statistical significance.

We can therefore have some confidence in the result but further research is very likely to change this estimate of effect.

Can the results of the research be applied to Ghana?

The trial was conducted in a low income setting in Nepal where the majority of women give birth at home without skilled assistance, and where exposure to environmental pathogens is high.

The interventions used were: 4% chlorhexidine (applied by a trained study worker during home visits on days 1, 2, 3, 4, 6, 8 & 10); soap and water (applied on the same days), and dry cord care (where mothers were visited on these days but only advice was given). Chlorhexidine was not compared to alcohol, which may be the most widely used method in Ghana.

In addition, all pregnant women received educational messages on hand-washing, sterile cord cutting, the importance of keeping the cord clean and avoiding applications of dung, mud or ash.

About quality of evidence (GRADE)

The GRADE system considers 'quality' to be a judgment of the extent to which we can be confident that the estimates of effect are correct. The level of 'quality' is judged on a 4-point scale. Evidence from randomized controlled studies is initially graded as HIGH and downgraded by one, two or three levels after full consideration of : any limitations in the design of the studies, the directness (or applicability) of the evidence, and the consistency and precision of the results.

High: Further research is very unlikely to change our confidence in the estimate of effect.

Moderate: Further research is likely to have an important impact on our confidence in the estimate of effect and may change the estimate.

Low: Further research is very likely to have an important impact on our confidence in the estimate of effect and is likely to change the estimate

Very low: We are very uncertain about the estimate.

Summary of findings table 1

Chlorhexidine 4% compared to dry cord care for neonatal cord care

Patient or population: Neonates
Settings: Low / middle income countries.

Outcomes	Illustrative comparative risks* (95% CI)		Relative effect (95% CI)	No of Participants (studies)	Quality of the evidence (GRADE)	Comments
	Assumed risk<br>**Dry cord care**	Corresponding risk<br>**Chlorhexidine**				
**Death**	**All**					
	**19 per 1000**	**15 per 1000**	**RR 0.78**<br>(0.57 to 1.07)	10,006<br>(1 study)	**Low**[1,2,3,4]	
	**If the intervention starts within 24 hours**					
	**22 per 1000**	**14 per 1000**<br>(10 to 21)	**RR 0.66**<br>(0.46 to 0.95)	10,016<br>(1 study)	**Moderate**[1,2,3]	
	**If the intervention starts after 24 hours**					
	**15 per 1000**	**15 per 1000**<br>(8 to 29)	**RR 1.02**<br>(0.54 to 1.92)	10,016<br>(1 study)	**Low**[1,2,3,4]	
**Cord infection: Defined as severe redness with pus**	**11 per 1000**	**3 per 1000**<br>(2 to 6)	**IRR 0.25**<br>(0.12 to 0.53)	10,006<br>(1 study)	**Moderate**[1,2,5]	
**Cord infection: Defined as moderate or severe redness**	**152 per 1000**	**103 per 1000**<br>(76 to 105)	**IRR 0.68**<br>(0.58 to 0.80)	9562<br>(1 study)	**Moderate**[1,2,5]	
**Adverse events**	**-**	**-**	**-**	10,016<br>(1 study)	-	Not reported[6]

*The basis for the **assumed risk** is the risk of death in the groups treated with dry cord care in the included trials. Under these trial conditions, the risk of death may be underestimated.
The **corresponding risk** (and its 95% confidence interval) is based on the assumed risk in the comparison group and the **relative effect** of the intervention (and its 95% CI).
CI: Confidence interval; **RR:** Risk ratio;

[1] This single trial was conducted in Nepal in a setting where a high proportion of mothers give birth at home without skilled assistance and where exposure to environmental pathogens is high.
[2] This trial adequately concealed allocation, but due to the nature of the interventions cannot be considered to be truly blinded.
[3] Downgraded by 1 under directness as this is a single study from Asia. Traditional birth practices and exposure to environmental pathogens may differ between settings.
[4] Downgraded by 1 under precision as the result is not statistically significant, but the 95% CI includes the possibility of a clinically important effect.
[5] Downgraded by 1 under study limitations as the people assessing the cord were the same as those administering the treatment.
[6] This trial does not comment on adverse events.

Q2. What is the potential public health impact of applying the results to Ghana?

Neonatal mortality in Ghana remains high at 27 per 1000 live births, and this is higher than the 19 per 1000 that was observed in the Nepal trial (UNICEF 2009).

If the same 22% relative risk reduction was achieved in Ghana, chlorhexidine cord care could reduce neonatal mortality to 21 per 1000 live births (95% CI 15 to 29). In 2009, UNICEF reports 766,000 live births in Ghana. Chlorhexidine cleansing therefore has the potential to prevent 4,596 of the 20682 neonatal deaths which occur each year (UNICEF 2009).

However, it must be borne in mind that this effect was achieved through an intensive schedule of post-natal visits, which may not currently be achievable in Ghana.

Q3. Is the current formulation suitable for introduction to Ghana?

Description of the formulation

**Route of administration:**	Topical
**Additional requirements:**	None
**Storage:**	Room temperature, in an opaque container away from Sunlight.
**Stability:**	Shelf-life of 20-24 months
**Transport:**	No special requirements

Is the introduction of this formulation feasible?

**Locally available manufacturers:**	None
**Ghana FDB Registration:**	None
**International manufacturers:**	Mission(India), Smart Pharmaceuticals(India)
**Suggested level of prescribing:**	All levels. Including home deliveries by trained and untrained birth attendants.
**Educational requirements:**	Minimal, as would replace similar practices such as application of alcohol.
**System requirements:**	None
**Any other concerns:**	None

Will the introduction of this formulation be acceptable to all stakeholders?

**Toxicity:**	Contact dermatitis has been reported with prolonged use.<br>There is some evidence of systemic absorption through skin especially in pre-term neonates. The significance of this is unknown.
**Appropriateness of formulation:**	Excellent
**Additional Stakeholders:**	Midwives, birth attendants
**National Guidelines:**	None currently
**International Guidelines**	WHO recommends the used of Chlorhexidine 5% for cord care if an antiseptic is to be used

Q. What are the resource implications?

What does this formulation cost?

	Formulation	Median	Minimum	Maximum
**IDPI Price Guide:**	Chlorhexidine gluconate 5% solution	0.0032/ml	0.0023/ml	0.0052/ml
**WHO Sources and prices 2nd edition:**	None listed		-	-

Is it cost-effective?

We searched the Economic Evaluations database within the Cochrane library for evaluations of chlorhexidine in the prevention of neonatal sepsis and cord infections but none were found.

About the NHS Economic Evaluations Database within the Cochrane Library

As healthcare resources are finite, information about both costs and effects are essential to making evidence-based decisions about competing healthcare interventions. But information about cost-effectiveness can be difficult to identify, appraise and interpret.
The NHS Economic Evaluation Database (EED) assists decision-makers by systematically identifying economic evaluations from around the world, appraising their quality, and highlighting their relative strengths and weaknesses.
The NHS Economic Evaluations Database is produced by the Centre for Reviews and Dissemination (CRD) at the University of York, UK.

References

- World Health Organization. Care of the umbilical cord: a review of the evidence. World Health Organization WHO/RHT/MSM/98.4 1998.
- Zupan J, Garner P, Omari AAA. Topical umbilical cord care at birth. *Cochrane Database of Systematic Reviews* 2004, Issue 3. Art. No.: CD001057. DOI: 10.1002/14651858.CD001057.pub2.
- Ghana Ministry of Health. Ghana Standard Treatment Guidelines. Sixth edition. 2010. Ghana National Drugs Programme. Available at: http://ghndp.org
- Ghana Ministry of Health. Ghana Essential Medicines List, Sixth edition. 2010. Ghana National Drugs Programme. Available at: http://ghndp.org
- Lawn JE, Cousens S, Zupan J. 4 million neonatal deaths: When? Where? Why? Lancet. 2005; 365: 891-900
- Russell AD, Path FRC. Chlorhexidine: Antibacterial action and bacterial resistance. Infection. 1986; 14(5): 212-15
- Blencowe H, Cousens S, Mullany LC, Lee ACC, Kerber K, Wall S, et al. Clean birth and postnatal care practices to reduce neonatal deaths from sepsis and tetanus: a systematic review and Delphi estimation of mortality effect. BMC Public Health. 2011; 11(Suppl 3):S11
- McClure EM, Armbruster D, Bartlett A, Biggar R, Carpenter J, Free MJ et al. The use of chlorhexidine to reduce maternal and neonatal mortality and morbidity in low-resource settings. International Journal of Gynaecology and Obstetetrics. 2007; 97(2): 89–94
- Mullany JC, Darmstadt GL, Tielsch JM. Safety and Impact of Chlorhexidine Antisepsis Interventions for Improving Neonatal Health in Developing Countries. Pediatric Infectious Diseases Journal. 2006; 25(8): 665–675
- Mullany LC, Darmstadt GL, Khatry SK, Katz J, LeClerq SC, Srestha S, et al. Topical applications of chlorhexidine to the umbilical cord for prevention of omphalitis and neonatal mortality in southern Nepal: a community-based, cluster-randomised trial. *Lancet*. 2006 March 18; 367(9514): 910–918.
- Mullany LC, El Arifeen S, Winch PJ, Shah R, Mannan I, Rahman SM. Impact of 4.0% chlorhexidine cleansing of the umbilical cord on mortality and omphalitis among newborns of Sylhet, Bangladesh: design of a community-based cluster randomized trial. BMC Pediatrics 2009; 9:67
- ZamCAT. Zambia Chlorhexidine Application Trial. Available at: http://www.bu.edu/cghd/projects/zambia-chlorhexidrine-application-trial-zamcat/
- Ghana Statistical Service (GSS), Ghana Health Service (GHS), and ICF Macro. 2009. Ghana Demographic and Health Survey 2008. Accra, Ghana: GSS, GHS, and ICF Macro.
- WHO Study on maternal mortality and neonatal morbidity in Africa Authors/Editors: Emmanuel Dzadeyson Publisher/Journal: Rural Integrated Relief Service-Ghana Publication date: 2007 http://www.who.int/pmnch/topics/health_systems/rirs_ghana/en/ accessed 27th August, 2011
- Garland JS, Alex CP, Mueller CD, et al. A randomized trial comparing povidone-iodine to a chlorhexidine gluconate-impregnated dressing for prevention of central venous catheter infections in neonates. Pediatrics 2001;107:1431–36. [PubMed: 11389271]
- http://www.unicef.org/infobycountry/ghana_statistics.html

Annex 1. Detailed search strategy and results

Set		Cochrane	PubMed
1		neonate OR newborn	chlorhexidine
2		cord OR chlorhexidine	neonate OR newborn
3		1 AND 2	1 AND 2
4			Limit 4 to review
**Search results**		**Cochrane**	**PubMed**
Hits		19	21
Included		1	3
Excluded		18	18
Reason for exclusion	Topic not relevant to this summary	17	18
	Not a systematic review	1 (protocol)	
	More complete reviews are available		
Additional reviews identified through reference lists			

Annex 2. Assessment of the risk of bias of a randomized controlled trial

Study reference: Mullany LC, Darmstadt GL, Khatry SK, Katz J, LeClerq SC, Srestha S, et al. Topical applications of chlorhexidine to the umbilical cord for prevention of omphalitis and neonatal mortality in southern Nepal: a community-based, cluster-randomised trial. *Lancet*. 2006 March 18; 367(9514): 910–918

Description of the study

**Study population:**	15,504 infants born between Nov 2002 and March 2005 in villages in rural Nepal
**Intervention:**	A non-medical project worker visited the newborn on days 1,2,3,4,6,8,10,12,14,21,28. The worker gave educational messages about clean cord care, then washed their hands, and cleansed the umbilical cord with 4% chlorhexidine.
**Control:**	1) As above except soap and water was used for cleaning<br>2) Health messages only (Dry cord care)
**Outcomes:**	Mortality, cord infections defined as 1) moderate or severe redness, 2) moderate or severe redness, with pus, or severe redness alone, 3) severe redness with pus.

Risk of Bias Criteria	Judgement
**1a. How did they generate a random sequence?**<br>*Examples of methods at low risk of selection bias: Computer randomisation, a random numbers table, shuffling cards, tossing a coin, drawing lots.*<br>'Clusters were randomized with a computerised random number generator'	☐ High risk of bias<br>☐ Unclear risk<br>☑ Low risk of bias
**1b. How did they conceal allocation?**<br>*Examples of methods at low risk of selection bias: Centralized or telephone allocation, sequentially numbered, sealed, opaque envelopes.*<br>'None described'	☐ High risk of bias<br>☑ Unclear risk<br>☐ Low risk of bias
**3. Were patients and study staff blinded to which treatment the participant received?**<br>*Examples of outcomes at low risk of performance bias: If the outcome is objective (eg. death) then blinding is less critical. If the outcome is subjective (e.g. symptoms or function) then blinding of the outcome assessor is critical.*<br>'The perfume used in the commercial cleanser was added to the chlorhexidine solution to make the smell of the solutions indistinguishable. The intervention solutions were packaged in identical opaque plastic bottles.'	☐ High risk of bias<br>☑ Unclear risk<br>☐ Low risk of bias
**4. Were outcome assessors blinded to which treatment the participant received?**<br>*Examples of outcomes at low risk of detection bias: If the outcome is objective (e.g. death) then blinding is less critical. If the outcome is subjective (e.g. symptoms or function) then blinding of the outcome assessor is critical.*<br>The same person who applied the solution assessed the umbilical cord. Probably high risk of bias for cord redness outcomes. Low risk of bias for death.	☑ High risk of bias<br>☐ Unclear risk<br>☐ Low risk of bias
**5. Have missing data due to participant withdrawals from the study been handled appropriately?**<br>*Examples of low risk of attrition bias: Withdrawals are low (less than 10%) and the reasons for withdrawal are clearly stated and balanced between groups.*<br>Dropouts were less than 5% in each group and balanced between groups	☐ High risk of bias<br>☐ Unclear risk<br>☑ Low risk of bias
**6. Is there evidence of selective outcome reporting?**<br>*Examples of low risk studies: If the protocol is available and all pre-specified outcomes appear in the report, or if given the nature of the question all expected outcomes have been reported.*<br>The protocol was not retrieved. The outcomes seem appropriate. It is unclear whether the sub-group analysis by time of first application was a pre-planned analysis or port-hoc.	☐ High risk of bias<br>☐ Unclear risk<br>☑ Low risk of bias
**7. Is there evidence of any other forms of bias?**<br>*Examples of other bias: One of the authors has a history of fraudulent reporting, stopping a trial early,*<br>No other bias identified.	☐ High risk of bias<br>☐ Unclear risk<br>☑ Low risk of bias

For further information on the Cochrane Tool for Assessing the Risk of Bias see:

Annex 3. Assessment of the local applicability of the trial (SUPPORT tool 9)

Study reference: Mullany LC, Darmstadt GL, Khatry SK, Katz J, LeClerq SC, Srestha S, et al. Topical applications of chlorhexidine to the umbilical cord for prevention of omphalitis and neonatal mortality in southern Nepal: a community-based, cluster-randomised trial. *Lancet*. 2006 March 18; 367(9514): 910–918

1. Were the studies included in this systematic review conducted in settings similar to Ghana, or were the findings consistent across settings and time periods?

Yes, the trial was conducted in a setting similar to Ghana; high birth rate and low socioeconomic status.

2. Are there important differences in on-the-ground realities and constraints in Ghana that might substantially alter the feasibility and acceptability of this drug/formulation?

There are no local manufacturers of chlorhexidine and may be importation may increase the price significantly.

3. Are there important differences in health system arrangements that may mean this drug/formulation could not work in the same way?

One important difference in health system arrangement is the fact that in Ghana, post-natal home visits are not done as in the case of the trial. However with adequate training of mothers, these home visits might not be required.

4. Are there important differences in the baseline conditions that might yield different absolute effects even if the relative effectiveness was the same?

The higher neonatal death rate in Ghana suggests that the absolute benefits may be greater than seen in Nepal.

5. What insights can be drawn about options, implementation, and monitoring and evaluation?

Its implementation would require adequate training of all stakeholders and mothers of the use of chlorhexidine with emphasis on the need to apply it on time.

For further information on the SUPPORT tool used for this assessment see: Lavis JN, Oxman AD, Souza NM, Lewin S, Gruen RL, Fretheim A. SUPPORT Tools for evidence-informed health Policymaking (STP) 9: Assessing the applicability of the findings of a systematic review. Health Res Policy Syst 2009, 7 Suppl 1:S9
