# Supplementary material for: Integrating Global and National Knowledge to Select Medicines for Children: The Ghana National Drugs Programme
Source: PLoS Med. 2013 May 21;10(5):e1001449. doi: 10.1371/journal.pmed.1001449 (PMC3660441; doi:10.1371/journal.pmed.1001449)
Supplement: Text S5 — Amoxicillin evidence summary. (PDF) [file pmed.1001449.s005.pdf]

Evidence Summary
For the Ghana Essential Medicines Committee

Title: **Dispersible amoxicillin tablets for children**

Formulation: Amoxicillin dispersible tablets (scored); 250mg and 500mg

Executive Summary;

Context: Dispersible paediatric tablets have many potential advantages over traditional suspensions and are now recommended by the World Health Organisation where available.
In Ghana, the first line treatment of non-severe pneumonia is currently seven days of oral amoxicillin, but the World Health Organization now recommend shorter courses.

Effects: **Advantages of short courses of antibiotics**

- 3 days of amoxicillin may be as effective as 5 days at reducing treatment failure (*low quality evidence*), and relapse (*low quality evidence*).
- Short courses probably improve compliance (*moderate quality evidence*)

Advantages of dispersible tablets compared to suspension

- Dose accuracy: Dosing of liquids by spoon is unreliable.
- Storage: Long shelf-life and no need for refridgeration.
- Transport: Less bulky and less susceptible to high temperatures.

Feasibility: A reliable and affordable supplier would need to be found.

Acceptability: Paediatric dispersible tablets are already in use in Ghana for malaria, and well tolerated.

Cost: Based on WHO price lists, dispersible tablets could represent a cost saving compared to suspensions.

Conclusion: Dispersible amoxicillin offers significant benefits over suspensions in terms of cost, product stability and ease of transport.
There is limited evidence to support a change in policy to recommend amoxicillin 25mg/kg twice daily for 3 to 5 days.

For consideration: Consider adding dispersible amoxicillin to the Ghana EML.

About this evidence summary

Who prepared this summary? This summary was prepared by Daniel Kwame Afriyie, Cynthia Amaning Danquah & Kwame Ohene Buabeng with technical support from the Liverpool School of Tropical Medicine.
Who funded this evidence summary? This evidence summary was funded by the Bill and Melinda Gates Foundation through the 'Better Medicines for Children Project', co-ordinated by the World Health Organization, in partnership with the Ghana Ministry of Health.
Declaration of conflicts of interest: None declared

Context

Why should this drug/formulation be considered by the committee?

Tablets and capsules can be difficult to swallow, and if perceived as bitter or unpleasant are often refused by children especially when ill (WHO 2008). To overcome these problems, many paediatric medicines are given as liquid suspensions or syrups, with the taste masked by fruity or sweet flavourings.

Effective drug administration in children requires formulations which allow for accurate weight or age- based dosing. With syrups and suspensions this is often done as full, half, or quarter spoonfuls, which has been shown to be inaccurate (Grießmann 2007). An alternative is to dispense measured syringes alongside medications.

In a position statement on the preferred dosage forms for use in children, the World Health Organization now recommend that where available dispersible tablets should be chosen above suspensions due to advantages in dosing, storage, and transportation (WHO 2009). Dispersible amoxicillin has been identified as a priority medicine for the treatment of pneumonia; formulated as scored tablets of 250mg and 500mg, and packaged in blister packs of 10 (WHO/UNICEF 2010a).

In addition, the Ghana STG currently recommends amoxicillin three-times daily for seven days for community acquired pneumonia (Ghana STG 2010). This may require revision in line with current WHO recommendations of 25mg/kg twice daily for 3 days in areas with low prevalence of HIV, and twice daily for 5 days in areas with a high prevalence of HIV (WHO 2003, Grant 2009).

Dispersible amoxicillin is not currently listed on the Ghana essential medicines list (Ghana EML, 2010).

What questions does this evidence summary aim to address?

This evidence summary aims to answer the following questions:

1. **Are twice daily regimens of amoxicillin for 3-5 days effective for treating pneumonia in children?**
2. **Are dispersible amoxicillin tablets well tolerated by young children?**
3. **Is a formulation suitable for Ghana currently available?**
4. **What are resource implications for this change?**

Effects

Q1. Are twice daily regimens of amoxicillin for 3-5 days effective at treating community acquired pneumonia in children?

What causes community acquired pneumonia and how might this regimen benefit?

Pneumonia is an infection of the lung tissue and is the leading cause of death in children aged less than 5 years. The common causes are respiratory viruses and bacteria such as S. pneumoniae, S. aureus and H. influenzae (Kabra 2010).

Most community acquired pneumonias are managed as outpatients with antibiotics, without microbiological confirmation of the infecting organism. The diagnosis is based on clinical signs with antibiotics recommended for children with a history of cough and fever and a respiratory rate above age-related cut-offs (Ghana STG 2010).

Amoxicillin is a well established first-line treatment for community acquired pneumonia with good activity against the common causes in children aged less than 5-years (Grant 2009). The traditional duration of treatment (commonly 7-10 days), does not have a strong evidence base (Haider 2011). Shorter courses may be cost saving and also encourage improved compliance (Grant 2009).

What research evidence is available?

The evidence base for the current WHO recommendations is summarized in two documents; WHO 2003 and Grant 2009.

In addition, we searched the Cochrane library for systematic reviews of antibiotics in the treatment of pneumonia using the search strategy outlined in Annex 1. We found two Cochrane reviews relevant to this question; Haider 2011 (up-to-date Sept 2010), & Kabra 2010 (up-to-date to Sept 2009).

What does the research show?

The WHO recommendation of 50mg twice-daily for 3-5 days is based on the findings of two trials included in the first Cochrane review (Haider 2011), which compared 3 days of amoxicillin with 5 days of amoxicillin (ISCAP 2004 and MASCOT 2004). These trials were conducted in children aged 2 to 59 months presenting with non-severe pneumonia in India and Pakistan. Both trials used regimens of three-times daily in both treatment arms. The move towards a twice regimen appears to be based on a single pharmacokinetic trial of 66 children (Fonseca 2003).

The second Cochrane review aimed to summarize the benefits and harms of different antibiotic regimens (including amoxicillin) for treating community acquired pneumonia (Kabra 2010). Twenty-seven trials were included of which 7 had a study arm which received amoxicillin, but only two used a twice daily dosing schedule for 5 days (CATCHUP 2002 and Hazir, 2008). The comparison groups in these trials received co-trimoxazole or ampicillin respectively. The characteristics of these four trials are shown in Annex 2.

The benefits of short courses of amoxicillin.

- ❖ We found no trials directly comparing amoxicillin given for 5 days versus amoxicillin for 7 days.
- ❖ 3 days of amoxicillin may be as effective as 5 days at reducing treatment failure (2 trials, 4012 participants, RR 1.11, 95% CI 0.94 to 1.33, *moderate quality evidence*).
- ❖ 3 days of amoxicillin may not result in more relapses than 5 days (2 trials, 3577 participants, RR 1.05, 95% CI 0.68 to 1.60, *moderate quality evidence*).
- ❖ Compliance over 3 days is probably better than compliance over 5 days (1 trial, 2188 participants, mean adherence over 3 days 94% vs over 5 days 85%, *moderate quality evidence*).

About systematic reviews

What is a systematic review? A systematic review seeks to answer a well formulated and specific question by identifying, critically appraising, and summarising the results of <u>all</u> relevant trials, published and unpublished, according to pre-stated and transparent methods.

What is a Cochrane Systematic Review? The Cochrane Collaboration is an international network of more than 28,000 people from over 100 countries. The collaboration is one of the biggest producers of systematic reviews on the effects of healthcare interventions, and Cochrane Systematic Reviews are recognized internationally as the benchmark for high quality information. Over 4,600 reviews have now been published online in *The Cochrane Library*. http://www.thecochranelibrary.com

What about non-Cochrane systematic reviews? Non-Cochrane reviews can be variable in quality. Important predictors of quality are: a broad and exhaustive search strategy, an assessment of the risk of bias of included studies, and freedom from conflicts of interest.

The benefits of amoxicillin twice daily instead of three times daily

- We found no trials directly comparing these two treatment regimens which gave efficacy data.
- The one pharmacokinetic study quoted by the WHO concluded that a twice daily regimen was a feasible alternative (Fonseca 2003). Other pharmacokinetic studies may be available but were not searched for as part of this evidence review.

Q2. Are oral dispersible tablets well tolerated in young children?

What research evidence is available?

We found no trials directly comparing orodispersible tablets with syrups and suspensions.

The only trial using oro-dispersible amoxicillin which we found compared 5-days three times daily regimen with that of 3-days (ISCAP 2004).

The benefits of dispersible amoxicillin instead of syrups or suspensions

- We found no trials directly comparing these two formulations.
- One trial enrolling 2188 children administered amoxicillin as oro-dispersible tablets (ISCAP 2004). Mean compliance was 84.9% for a 5-day course. No other comment on tolerability was made.

Are the results of the research reliable?

How much confidence can we have in the systematic review methods?

The Cochrane reviews were well conducted with only minor limitations (see Annex 3&4). They conducted extensive searches of The Cochrane Library, Medline, EMBASE, and LILACS and are unlikely to have missed major randomised controlled trials of amoxicillin in the treatment of childhood pneumonia. Trials of 5 days versus 7 days and twice-daily versus three-times daily would have been included if they existed.

Can the results of the research be applied to Ghana?

The trials used standard WHO criteria for diagnosing clinical pneumonia.

All the trials were conducted in Asia and both the common infecting organisms and antibacterial resistance patterns may differ between these settings and Ghana.

Although there are no trials directly comparing twice daily amoxicillin with three times daily, or five days of amoxicillin with seven days, there is some indirect evidence that a twice daily regimen for 3 to 5 days may be equally effective.

About quality of evidence (GRADE)

The GRADE system considers 'quality' to be a judgment of the extent to which we can be confident that the estimates of effect are correct. The level of 'quality' is judged on a 4-point scale. Evidence from randomized controlled studies is initially graded as HIGH and downgraded by one, two or three levels after full consideration of : any limitations in the design of the studies, the directness (or applicability) of the evidence, and the consistency and precision of the results.

High: Further research is very unlikely to change our confidence in the estimate of effect.
Moderate: Further research is likely to have an important impact on our confidence in the estimate of effect and may change the estimate.
Low: Further research is very likely to have an important impact on our confidence in the estimate of effect and is likely to change the estimate
Very low: We are very uncertain about the estimate.

Summary of findings table

5 days treatment with amoxicillin versus 3 days treatment for non-severe pneumonia

Patient or population: patients with non-severe pneumonia
Settings: Low and middle income countries
Intervention: 5 days treatment versus 3 days treatment

Outcomes	Illustrative comparative risks* (95% CI)		Relative effect (95% CI)	No of Participants (studies)	Quality of the evidence (GRADE)	Comments
	Assumed risk	Corresponding risk				
	**5 days treatment**	**3 days treatment**				
**Treatment failure at day 5**	**103 per 1000**	**114 per 1000** (96 to 136)	**RR 1.11** (0.94 to 1.33)	4012 (2 studies)	**low**[1,2,3]	
**Relapse rate**	**23 per 1000**	**25 per 1000** (16 to 37)	**RR 1.05** (0.69 to 1.6)	3577 (2 studies)	**low**[1,2,3]	
**Compliance**				2188 (1 study)	**moderate**[4,5]	

*The basis for the **assumed risk** is provided in footnotes. The **corresponding risk** (and its 95% confidence interval) is based on the assumed risk in the comparison group and the **relative effect** of the intervention (and its 95% CI).
CI: Confidence interval; **RR:** Risk ratio;

GRADE Working Group grades of evidence
High quality: Further research is very unlikely to change our confidence in the estimate of effect.
Moderate quality: Further research is likely to have an important impact on our confidence in the estimate of effect and may change the estimate.
Low quality: Further research is very likely to have an important impact on our confidence in the estimate of effect and is likely to change the estimate.
Very low quality: We are very uncertain about the estimate.

[1] Both trials were well conducted and considered at low risk of bias.
[2] Downgraded by 1 for indirectness: These two studies were conducted in India and Pakistan in children aged 2 to 59 months presenting with clinical signs of non-severe pneumonia. Given the potential differences in prevalence of organisms and resistance the results may not be generalisable to African settings.
[3] Downgraded by 1 for imprecision. The 95% CI includes the possibility of a clinically important increase in treatment failure with 3 days amoxicillin.
[4] Of these 2 trials only ISCAP 2004 reported on adherence at day 3 and day 5.
[5] Down graded by 1 for indirectness as neither group in this trial received the current recommended schedule of 50mg twice daily for 3-5 days.
[6] The mean adherence was 94% at 3 days and 85% at 5 days. Statistical significance not reported

Q3. Is the current formulation suitable for introduction to Ghana?

Description of the formulation

	Dispersible tablets	Suspension
**Route:**	Oral	Oral
**Preparation:**	Only require small volumes of fluid	Can be difficult to ensure an even volume and sustained suspension.
**Palatability:**	Dependant on formulation	Dependant on formulation
**Dose accuracy:**	More certain, as each dose requires the complete intake of one or half tablet	Can be uncertain due to inaccuracy of spoon measurement (Grießmann 2007), or suspensions settling or being incompletely dissolved
**Storage:**	Tablets could be stored at room temperature.	Dry powder may be stored at room temperature, but once reconstituted requires refrigeration at 2-8°C for not more than 14 days.
**Stability:**	Relatively Long shelf-life compared to reconstituted syrups/suspensions	Shorter shelf life compared to the dispersible tablets
**Transport:**	May be easier and cheaper due to smaller volume	Bulky

Is the introduction of this formulation feasible?

Locally available manufacturers: None.

Ghana FDB Registration: One listed: Amoxi-Denk 250mg; Registered by Denk Pharma-Germany.

International manufacturers: Five listed in WHO sources and prices 2nd edition:
- Athlone (Ireland),
- Esco(India),
- Microlabs (India),
- Hovid (Malaysia),
- Remedica (Cyprus)

Suggested level of prescribing: D, C, B2, B1

Educational requirements: Minimal as other dispersible pediatric tablets are already in use (e.g. artemether-lumefantrine).

System requirements: None

Any other concerns: None

Will the introduction of this formulation be acceptable to all stakeholders?

Toxicity: As for amoxicillin suspension

Appropriateness of formulation: Scored 250mg tablets allow convenient weight or age based dosing

Additional Stakeholders: National Health Insurance Authority, Prescribers, Pharmacists,

National Guidelines: Amoxicillin is a first-line antibiotic for pneumonia and otitis media. This formulation is not currently listed on the Ghana EML or NHIL

International Guidelines This formulation is listed on the WHO model EDL

Q. What are the resource implications?

What does this formulation cost?

		Median	Minimum	Maximum
**IDPI Price Guide:**				
**Dispersible tablet 250mg**	None listed	-	-	-
	Per 5-day course (10 tablets)			
**Powder for oral suspension 250mg/5ml**	Per ml	$ 0.006	-	-
	Per 5-day course (100 ml)	$ 0.6	-	-
WHO Sources and prices 2nd edition:				
**Dispersible tablet 250mg**	Per tablet	$ 0.022	$ 0.017	$ 0.023
	Per 5-day course (10 tablets)	$ 0.22	$ 0.17	$ 0.23
**Powder for oral suspension 250mg/5ml**	Per ml	$ 0.008	$ 0.002	$ 0.058
	Per 5-day course (100 ml)	$ 0.8	$ 0.2	$ 5.8

Is it cost-effective?

We searched the Economic Evaluation Database within the Cochrane library for cost analyses of dispersible amoxicillin and did not find any.

The prices quoted for dispersible amoxicillin suggest that dispersible tablets could represent a cost saving over suspension if a reliable supplier can be found: cost per treatment course; $ 0.22 dispersible tablets vs $ 0.8 suspension (WHO 2010b).

About the NHS Economic Evaluations Database within the Cochrane Library

As healthcare resources are finite, information about both costs and effects are essential to making evidence-based decisions about competing healthcare interventions. But information about cost-effectiveness can be difficult to identify, appraise and interpret.

The NHS Economic Evaluation Database (EED) assists decision-makers by systematically identifying economic evaluations from around the world, appraising their quality, and highlighting their relative strengths and weaknesses.

The NHS Economic Evaluations Database is produced by the Centre for Reviews and Dissemination (CRD) at the University of York, UK.

References

- WHO 2008. The pathway to better medicines for children. 2008. Available at: http://www.who.int/childmedicines/Pathway.pdf
- Grießmann K, Breitkreutz J, Schubert-Zsilavecz et al. Dosing accuracy of measuring devices provided with antibiotic oral suspensions. *Paediatric and Perinatal Drug Therapy.* 2007; **8:** 61-70.
- *WHO technical report series; 958. The selection and use of essential medicines: report of the WHO Expert Committee, March 2009*. Geneva, World Health Organization, 2009. Available at:
- WHO/UNICEF 2010a. Priority essential medicines for child survival. 2010. Available at: http://www.who.int/childmedicines/progress/Unicef_prioriry_meds_child_survival.pdf
- Ghana Ministry of Health. Ghana Standard Treatment Guidelines. Sixth edition. 2010. Ghana National Drugs Programme. Available at: http://ghndp.org
- Ghana Ministry of Health. Ghana Essential Medicines List, Sixth edition. 2010. Ghana National Drugs Programme. Available at: http://ghndp.org
- Kabra SK, Lodha R, Pandey RM. Antibiotics for community-acquired pneumonia in children. Cochrane Database of Systematic Reviews 2010, Issue 3. Art. No.: CD004874. DOI: 10.1002/14651858.CD004874.pub3.
- Grant GB, Campbell H, Dowell SF, Graham SM, Klugman KP, Mulholland EM, et al. Recommendations for treatment of childhood non-severe pneumonia. Lancet Infectious Diseases. 2009; 9: 185–96.
- Haider BA, Lassi ZS, Bhutta ZA. Short-course versus long-course antibiotic therapy for non-severe community-acquired pneumonia in children aged 2 months to 59 months. *Cochrane Database of Systematic Reviews* 2008, Issue 2. Art. No.: CD005976. DOI: 10.1002/14651858.CD005976.pub2.
- WHO. Consultative meeting to review evidence and research priorities in the management of acute respiratory infections (ARI). Geneva, September 29–October 1, 2003. Meeting report [WHO/FCH/CAH/04.2]. Available at: http://whqlibdoc.who.int/hq/2004/WHO_FCH_CAH_04.2.pdf
- Fonseca W, Hoppu K, Rey LC, Amaral J, Qazi S. Comparing pharmacokinetics of amoxicillin given twice or three times per day to children older than 3 months with pneumonia. Antimicrobial Agents Chemotherapy. 2003;47:997-1001
- CATCHUP Study Group. Clinical efficacy of cotrimoxazole versus amoxicillin twice daily for treatment of pneumonia: a randomized controlled clinical trial in Pakistan. *Archives of Diseases in Childhood.* 2002;**86**:113–8.
- Hazir T, Fox LM, Nisar YB, Fox MP, Ashraf YP, MacLeod WB, et al. Ambulatory short-course high-dose oral amoxicillin for treatment of severe pneumonia in children: a randomised equivalency trial. *Lancet* 2008;**371**:49-56.
- ISCAP Study Group. Three day versus five day treatment with amoxicillin for non-severe pneumonia in young children: a multicentre randomised controlled trial. *BMJ* 2004;**328**(7443)
- MASCOT. Clinical efficacy of 3 days versus 5 days of oral amoxicillin for treatment of childhood pneumonia: a multicentre double-blind trial. *Lancet* 2002;**360**(9336):835–41.
- Dhaon Nitin (2004), Amoxicillin tablets for oral suspension in the treatment of acute otitis media: a new formulation with improved convenience. Advances in therapy., 21(2) 87-94
- www.unicef.org/infobycountry/ghana_statistics.html
- WHO/UNICEF 2010. Sources and prices of selected medicines for children including therapeutic food, dietary vitamin and mineral supplementation. 2010. Second edition. Available at: http://www.who.int/medicines/publications/essentialmedicines/Sources_Prices2010.pdf
- MSH 2010. International Drug Price Indicator Guide. 2010. Available at: http://erc.msh.org/mainpage.cfm?file=1.0.htm&id=1&temptitle=Introduction&module=DMP&language=English

Annex 1. Detailed search strategy and results

Set		Cochrane
1		amoxicillin
2		pneumonia
3		1 AND 2
4		
**Search results**		**Cochrane**
Hits		5
Included		2
Excluded		1
Reason for exclusion	Topic not relevant to this summary	3
	Not a systematic review	
	More complete reviews are available	
Additional reviews identified through reference lists		

Annex 2. Summary of trials of amoxicillin for 5-days in the treatment of pneumonia in children

Study details				Amoxicillin				Comparator			
Study ID	Country	Diagnosis	Age (m)	Route	Formulation	Dose	Treatment failure	Drug	Route	Dose	Treatment failure
CATCHUP 2002	Pakistan	Non-severe pneumonia	2 to 59	Oral	Not stated	25mg/kg BD for 5 days	117/725 (16%)	Co-trimoxazole	Oral	4:20mg/kg BD for 5 days	139/734 (19%)
Hazir 2008	Pakistan	Severe pneumonia	3 to 59	Oral	Syrup	45mg/kg BD for 5 days	77/1025 (8%)	Ampicillin	IV/IM	QDS for 48 hrs Then oral for 3 days	87/1012 (9%)
ISCAP 2004	India	Non-severe pneumonia	2 to 59	Oral	Dispersible	125 mg TDS for 5 days	43/1026 (4%)	Amoxicillin	Oral	125 mg TDS for 3 days	53/1033 (5%)
MASCOT 2004	Pakistan	Non-severe pneumonia	2 to 59	Oral	Not stated	15 mg/kg TDS for 5 days	162/973 (17%)	Amoxicillin	Oral	15 mg/kg TDS for 3 days	177/980 (18%)

Annex 3. AMSTAR assessment of the systematic review

Review reference: Haider BA, Lassi ZS, Bhutta ZA. Short-course versus long-course antibiotic therapy for non-severe community-acquired pneumonia in children aged 2 months to 59 months. *Cochrane Database of Systematic Reviews* 2008, Issue 2. Art. No.: CD005976. DOI: 10.1002/14651858.CD005976.pub2

1. Was an 'a priori' design provided?
The research question and inclusion criteria should be established before the conduct of the review.

☑ Yes
☐ No
☐ Can't answer
☐ Not applicable

2. Was there duplicate study selection and data extraction?
There should be at least two independent data extractors and a consensus procedure for disagreements should be in place.

☑ Yes
☐ No
☐ Can't answer
☐ Not applicable

3. Was a comprehensive literature search performed?
At least two electronic sources should be searched. The report must include years and databases used (e.g. Central, EMBASE, and MEDLINE). Key words and/or MESH terms must be stated and where feasible the search strategy should be provided. All searches should be supplemented by consulting current contents, reviews, textbooks, specialized registers, or experts in the particular field of study, and by reviewing the references in the studies found.

☑ Yes
☐ No
☐ Can't answer
☐ Not applicable

4. Was the status of publication (i.e. grey literature) used as an inclusion criterion?
The authors should state that they searched for reports regardless of their publication type. The authors should state whether or not they excluded any reports (from the systematic review), based on their publication status, language etc.

☐ Yes
☑ No
☐ Can't answer
☐ Not applicable

5. Was a list of studies (included and excluded) provided?
A list of included and excluded studies should be provided.

☑ Yes
☐ No
☐ Can't answer
☐ Not applicable

6. Were the characteristics of the included studies provided?
In an aggregated form such as a table, data from the original studies should be provided on the participants, interventions and outcomes. The ranges of characteristics in all the studies analyzed e.g. age, race, sex, relevant socioeconomic data, disease status, duration, severity, or other diseases should be reported.

☑ Yes
☐ No
☐ Can't answer
☐ Not applicable

7. Was the scientific quality of the included studies assessed and documented?
'A priori' methods of assessment should be provided (e.g., for effectiveness studies if the author(s) chose to include only randomized, double-blind, placebo controlled studies, or allocation concealment as inclusion criteria); for other types of studies alternative items will be relevant.

☑ Yes
☐ No
☐ Can't answer
☐ Not applicable

8. Was the scientific quality of the included studies used appropriately in formulating conclusions?
The results of the methodological rigor and scientific quality should be considered in the analysis and the conclusions of the review, and explicitly stated in formulating recommendations.

☐ Yes
☐ No
☐ Can't answer
☐ Not applicable

9. Were the methods used to combine the findings of studies appropriate?
For the pooled results, a test should be done to ensure the studies were combinable, to assess their homogeneity (i.e. Chi-squared test for homogeneity, I^2). If heterogeneity exists a random effects model should be used and/or the clinical appropriateness of combining should be taken into consideration (i.e. is it sensible to combine?).

☑ Yes
☐ No
☐ Can't answer
☐ Not applicable

10. Was the likelihood of publication bias assessed?
An assessment of publication bias should include a combination of graphical aids (e.g., funnel plot, other available tests) and/or statistical tests (e.g., Egger regression test).

☐ Yes
☑ No
☐ Can't answer
☐ Not applicable

11. Was the conflict of interest stated?
Potential sources of support should be clearly acknowledged in both the systematic review and the included studies.

☑ Yes
☐ No
☐ Can't answer
☐ Not applicable

For further information on the AMSTAR tool see: Shea B, Grimshaw J, Wells G, Boers M, Andersson N, Hamel C, et al. Development of AMSTAR: a measurement tool to assess the methodological quality of systematic reviews. BMC Medical Research Methodology. 2007; 7(1):10.

Annex 4. AMSTAR assessment of the systematic review

Review reference: Kabra SK, Lodha R, Pandey RM. Antibiotics for community-acquired pneumonia in children. Cochrane Database of Systematic Reviews 2010, Issue 3. Art. No.: CD004874. DOI: 10.1002/14651858.CD004874.pub3

**1. Was an 'a priori' design provided?**<br>The research question and inclusion criteria should be established before the conduct of the review.	☑ Yes<br>☐ No<br>☐ Can't answer<br>☐ Not applicable
**2. Was there duplicate study selection and data extraction?**<br>There should be at least two independent data extractors and a consensus procedure for disagreements should be in place.	☑ Yes<br>☐ No<br>☐ Can't answer<br>☐ Not applicable
**3. Was a comprehensive literature search performed?**<br>At least two electronic sources should be searched. The report must include years and databases used (e.g. Central, EMBASE, and MEDLINE). Key words and/or MESH terms must be stated and where feasible the search strategy should be provided. All searches should be supplemented by consulting current contents, reviews, textbooks, specialized registers, or experts in the particular field of study, and by reviewing the references in the studies found.	☑ Yes<br>☐ No<br>☐ Can't answer<br>☐ Not applicable
**4. Was the status of publication (i.e. grey literature) used as an inclusion criterion?**<br>The authors should state that they searched for reports regardless of their publication type. The authors should state whether or not they excluded any reports (from the systematic review), based on their publication status, language etc.	☐ Yes<br>☑ No<br>☐ Can't answer<br>☐ Not applicable
**5. Was a list of studies (included and excluded) provided?**<br>A list of included and excluded studies should be provided.	☑ Yes<br>☐ No<br>☐ Can't answer<br>☐ Not applicable
**6. Were the characteristics of the included studies provided?**<br>In an aggregated form such as a table, data from the original studies should be provided on the participants, interventions and outcomes. The ranges of characteristics in all the studies analyzed e.g. age, race, sex, relevant socioeconomic data, disease status, duration, severity, or other diseases should be reported.	☑ Yes<br>☐ No<br>☐ Can't answer<br>☐ Not applicable
**7. Was the scientific quality of the included studies assessed and documented?**<br>'A priori' methods of assessment should be provided (e.g., for effectiveness studies if the author(s) chose to include only randomized, double-blind, placebo controlled studies, or allocation concealment as inclusion criteria); for other types of studies alternative items will be relevant.	☑ Yes<br>☐ No<br>☐ Can't answer<br>☐ Not applicable
**8. Was the scientific quality of the included studies used appropriately in formulating conclusions?**<br>The results of the methodological rigor and scientific quality should be considered in the analysis and the conclusions of the review, and explicitly stated in formulating recommendations.	☑ Yes<br>☐ No<br>☐ Can't answer<br>☐ Not applicable
**9. Were the methods used to combine the findings of studies appropriate?**<br>For the pooled results, a test should be done to ensure the studies were combinable, to assess their homogeneity (i.e. Chi-squared test for homogeneity, I^2). If heterogeneity exists a random effects model should be used and/or the clinical appropriateness of combining should be taken into consideration (i.e. is it sensible to combine?).	☑ Yes<br>☐ No<br>☐ Can't answer<br>☐ Not applicable
**10. Was the likelihood of publication bias assessed?**<br>An assessment of publication bias should include a combination of graphical aids (e.g., funnel plot, other available tests) and/or statistical tests (e.g., Egger regression test).	☑ Yes<br>☐ No<br>☐ Can't answer<br>☐ Not applicable
**11. Was the conflict of interest stated?**<br>Potential sources of support should be clearly acknowledged in both the systematic review and the included studies.	☑ Yes<br>☐ No<br>☐ Can't answer<br>☐ Not applicable

For further information on the AMSTAR tool see: Shea B, Grimshaw J, Wells G, Boers M, Andersson N, Hamel C, et al. Development of AMSTAR: a measurement tool to assess the methodological quality of systematic reviews. BMC Medical Research Methodology. 2007; 7(1):10.

Annex 5. Assessment of the local applicability of the systematic review (SUPPORT tool 9)

Review reference:

1. Were the studies included in this systematic review conducted in settings similar to Ghana, or were the findings consistent across settings and time periods?

2. Are there important differences in on-the-ground realities and constraints in Ghana that might substantially alter the feasibility and acceptability of this drug/formulation?

3. Are there important differences in health system arrangements that may mean this drug/formulation could not work in the same way?

4. Are there important differences in the baseline conditions that might yield different absolute effects even if the relative effectiveness was the same?

5. What insights can be drawn about options, implementation, and monitoring and evaluation?

For further information on the SUPPORT tool used for this assessment see: Lavis JN, Oxman AD, Souza NM, Lewin S, Gruen RL, Fretheim A. SUPPORT Tools for evidence-informed health Policymaking (STP) 9: Assessing the applicability of the findings of a systematic review. Health Res Policy Syst 2009, 7 Suppl 1:S9
