# Supplementary material for: Integrating Global and National Knowledge to Select Medicines for Children: The Ghana National Drugs Programme
Source: PLoS Med. 2013 May 21;10(5):e1001449. doi: 10.1371/journal.pmed.1001449 (PMC3660441; doi:10.1371/journal.pmed.1001449)
Supplement: Text S6 — Caffeine evidence summary. (PDF) [file pmed.1001449.s006.pdf]

Evidence Summary
For the Ghana Essential Medicines Committee

Title: **Caffeine Citrate for treating apnoea in preterm infants**

Formulation:
1. *Injection: 20 mg/ml (equivalent to 10 mg caffeine base/ml)*
2. *Oral liquid: 20 mg/ml (equivalent to 10 mg caffeine base/ml)*

Executive Summary

Context: The methylxanthines (aminophylline, theophylline and caffeine) have been widely used for many years to prevent and treat apnoea in premature neonates. However, evidence of the long-term benefits and harms has only recently become available. Caffeine Citrate is now recommended by the WHO for neonatal care, but is not listed on the Ghana STG, EML or NHIL

Effects: **Benefits of treating premature neonates with caffeine citrate**

- Probably reduces the risk of death or major disability by late infancy *(moderate quality evidence)*,
- Reduces the risk of chronic lung disease *(high quality evidence)*,
- Probably reduces the risk of cognitive delay *(moderate quality evidence)*,
- Probably reduces side effects compared to theophylline *(moderate quality evidence)*.

Harms of treating premature neonates with caffeine citrate

- None confirmed

Feasibility: There are currently no suitable products listed in either the IDPI price guide or the WHO sources and prices of medicines

Acceptability: No concerns

Cost: No prices or economic evaluations were available

Conclusion: Caffeine appears to have significant benefits in premature neonates.

For consideration: Addition of caffeine to the Ghana EML/NHIL
Identification of a supplier of an appropriate formulation, and estimation of the resources required.
National guidance for the use of caffeine.

About this evidence summary

Who prepared this summary? This summary was prepared by Benedicta Adwoa Yeboah & Priscilla Owusu-Ansah with technical support from the Liverpool School of Tropical Medicine.

Who funded this evidence summary? This evidence summary was funded by the Bill and Melinda Gates Foundation through the 'Better Medicines for Children Project', co-ordinated by the World Health Organization, in partnership with the Ghana Ministry of Health.
Declaration of conflicts of interest: None declared

Context

Why should this drug/formulation be considered by the committee?

The methylxanthines (aminophylline, theophylline and caffeine) have been widely used for many years to prevent and treat apnoea in premature neonates (Martin 2004). However, evidence of the long-term benefits and harms has only recently become available.

Caffeine citrate is now included on the World Health Organization model essential medicines list for children (WHO EML 2011). However, there is currently no paediatric formulation on the essential medicines list or on the national health insurance list in Ghana (Ghana EML, Ghana NHIL).

What questions does this evidence summary aim to address?

This evidence summary aims to answer the following questions:

1. **Does caffeine reduce the risk of death or long-term neurological disability when used to treat neonatal apnoea?**
2. **What could be the public health impact of the introduction of caffeine for neonatal care in Ghana?**
3. **Is there a formulation that is acceptable and feasible for introduction in Ghana?**
4. **What are the resource implications of this change?**

Effects

Q1. Is Caffeine Citrate superior to placebo for treating apnoea?

What is Apnoea and how might caffeine citrate work?

Recurrent apneas, defined as pauses in breathing lasting greater than 20 seconds, are commonly seen in infants born before 34 weeks gestation (Comer 2001). They are often associated with periods of hypoxia and bradycardia, which may have long term consequences for the developing brain, or other vital organs vulnerable to hypoxia (Martin 2004).

The methylxanthines (aminophylline, theophylline and caffeine), are respiratory stimulants, that are widely used to treat or prevent apnea. Where these fail, and apnoeic episodes are frequent or severe, these infants often require intubation and mechanical ventilation (Martin 2004).

What research evidence is available?

In July 2011, we searched the Cochrane Library and PubMed for systematic reviews evaluating caffeine citrate for the treatment of apnoea in pre-term infants. We found one review comparing any methylxanthine with placebo (Henderson-Smart 2010a), and one review directly comparing theophylline and caffeine (Henderson-Smart 2010b).

These two reviews were up-to-date to July 2010, and August 2009 respectively.

What does the research show?

The two reviews aimed to summarize the benefits and harms of caffeine citrate for treating apnoea in preterm infants. Three randomized controlled trials have compared caffeine to placebo; including one large trial enrolling 2006 infants (CAP Trial 2006), and two small trials enrolling 105 infants. Five very small trials have directly compared caffeine with theophylline; enrolling 108 infants.

Only one study evaluated aminophylline for treating neonatal apnoea, and this trial only reported outcomes at 48 hours.

The benefits of using caffeine citrate to treat apnea:
Compared to placebo:

- ❖ Caffeine probably reduces the risk of death or major disability by late infancy by 15% (RR 0.85, 95% CI 0.71 to 1.01, 767 participants, 1 study, *moderate quality evidence*),
- ❖ Caffeine probably reduces the risk of cerebral palsy by 40% (RR 0.60, 95% CI 0.29 to 1.25, 729 participants, 1 study, *moderate quality evidence*), and cognitive delay by 14% (RR 0.86, 95% CI 0.69 to 1.06, 715 participants, 1 study, *moderate quality evidence*)
- ❖ Caffeine reduces the risk of chronic lung disease by 38% (RR 0.72, 95% CI 0.58 to 0.89; 805 participants, 1 study, *high quality evidence*), and the risk of needing PDA ligation by 63% (RR 0.37, 95% CI 0.21 to 0.66; 827 participants, 1 study, *high quality evidence*).
- ❖ It is unknown whether caffeine reduces the need for mechanical ventilation as only one very small trial reported this outcome (18 participants, 1 study, *very low quality evidence*).
- ❖ Caffeine probably reduces the risk of continued apnea after 2-7 days (RR 0.46, CI 0.27 to 0.78; 100 participants, 2 studies, *moderate quality evidence*).

Compared to theophylline:

- ❖ Caffeine probably causes fewer side effects such as tachycardia or feed intolerance (RR 0.17 , 95% CI 0.04 to 0.72, 66 participants, 3 trials, *moderate quality evidence*)

The harms of using caffeine citrate to treat apnea:

- ❖ One trial reported a higher risk of dying before discharge with caffeine but the trial was too small to have any confidence in the result (82 participants, one study, *very low quality evidence*).

About systematic reviews

What is a systematic review? A systematic review seeks to answer a well formulated and specific question by identifying, critically appraising, and summarising the results of all relevant trials, published and unpublished, according to pre-stated and transparent methods.
What is a Cochrane Systematic Review? The Cochrane Collaboration is an international network of more than 28,000 people from over 100 countries. The collaboration is one of the biggest producers of systematic reviews on the effects of healthcare interventions, and Cochrane Systematic Reviews are recognized internationally as the benchmark for high quality information. Over 4,600 reviews have now been published online in *The Cochrane Library*. http://www.thecochranelibrary.com
What about non-Cochrane systematic reviews? Non-Cochrane reviews can be variable in quality. Important predictors of quality are: a broad and exhaustive search strategy, an assessment of the risk of bias of included studies, and freedom from conflicts of interest.

Are the results of the research reliable?

How much confidence can we have in the systematic review methods?

The Cochrane reviews were well conducted with only minor limitations (see Annex 2). An extensive search was conducted of the CENTRAL, MEDLINE, EMBASE, LILACS and CINAHL databases. It is unlikely that published trials assessing this question have been missed. The authors also searched for unpublished trials by reviewing trial registers and conference proceedings but none were found.

How much confidence can we have in the systematic review results?

The quality of the evidence provided by the Cochrane review has been assessed using the methods developed by the GRADE working group. A summary of the main results of the review, and the quality assessments are shown overleaf in the Summary of Findings table.

The evidence for the primary outcome of a reduction in death or severe disability was graded moderate as it did not quite reach statistical significance. This means that we can have a reasonable level of confidence in the result although further research may help. It should however be noted that this is a composite outcome and there was no evidence of a reduction in death when examined alone.

Can the results of the research be applied to Ghana?

The majority of data, particularly for long-term outcomes, is from a single multi-centre study (CAP Trial 2006) conducted in the USA, Europe, Canada, Australia and Israel. Although these settings have many differences to Ghana it is probably reasonable to assume that apnoea affects pre-term neonates in the same way around the world, and that in the absence of local research these results could reasonably be applied.

The CAP study included preterm infants with birth weights of 500 to 1250 grams. These infants require intensive care at specialist neonatal units, and so the use of caffeine should probably be limited to regional or large district hospitals within Ghana.

The caffeine dose used in the large CAP trial was 20 mg per kg IV, followed by a daily maintenance dose of 5 mg per kg. The daily maintenance dose could be given orally and also increased to 10 mg caffeine citrate per kg with the persistence of the apnoea condition.

About quality of evidence (GRADE)

The GRADE system considers 'quality' to be a judgment of the extent to which we can be confident that the estimates of effect are correct. The level of 'quality' is judged on a 4-point scale. Evidence from randomized controlled studies is initially graded as HIGH and downgraded by one, two or three levels after full consideration of : any limitations in the design of the studies, the directness (or applicability) of the evidence, and the consistency and precision of the results.

High: Further research is very unlikely to change our confidence in the estimate of effect.

Moderate: Further research is likely to have an important impact on our confidence in the estimate of effect and may change the estimate.

Low: Further research is very likely to have an important impact on our confidence in the estimate of effect and is likely to change the estimate

Very low: We are very uncertain about the estimate.

Summary of findings table

Caffeine citrate compared to placebo for treating apnoea

Patient or population: Preterm neonates
Settings: Premature neonates in low and middle income countries

Outcomes	Illustrative comparative risks* (95% CI)		Relative effect (95% CI)	No of Participants (studies)	Quality of the evidence (GRADE)	Comments
	Assumed risk<br>**Placebo**	Corresponding risk<br>**Caffeine Citrate**				
**Death or major disability before 18 months**	**417 per 1000**	**354 per 1000**<br>(296 to 421)	**RR 0.85**<br>(0.71 to 1.01)	767<br>(1 study)	**moderate**[1,2,3]	
**Death prior to hospital discharge**	**27 per 1000**	**44 per 1000**<br>(4 to 471)	**RR 1.64**<br>(0.16 to 17.43)	82<br>(1 study)	**very low**[4]	
**Cerebral palsy before 18 months**	**50 per 1000**	**30 per 1000**<br>(14 to 62)	**RR 0.6**<br>(0.29 to 1.25)	729<br>(1 study)	**moderate**[1,3]	
**Cognitive delay**	**343 per 1000**	**295 per 1000**<br>(237 to 364)	**RR 0.86**<br>(0.69 to 1.06)	715<br>(1 study)	**moderate**[1,3]	
**Chronic Lung Disease**	**359 per 1000**	**259 per 1000**<br>(208 to 230)	**RR 0.72**<br>(0.58 to 0.89)	805<br>(1 study)	**high**[1,5]	
**PDA ligation**	**100 per 1000**	**37 per 1000**<br>(21 to 66)	**RR 0.37**<br>(0.21 to 0.66)	827<br>(1 study)	**high**[1,5]	
**Need for Mechanical Ventilation**	**222 per 1000**	**44 per 1000**<br>(2 to 813)	**RR 0.20**<br>(0.01 to 3.66)	18<br>(1 study)	**very low**[4]	
**Failed apnoea reduction after 2-7 days**	**565 per 1000**	**260 per 1000**<br>(153 to 441)	**RR 0.46**<br>(0.27 to 0.78)	100<br>(2 studies)	**moderate**[6]	

*The basis for the **assumed risk** is the risk of the outcome in the groups treated with placebo in the included trials.
The **corresponding risk** (and its 95% confidence interval) is based on the assumed risk in the comparison group and the **relative effect** of the intervention (and its 95% CI).
CI: Confidence interval; **RR:** Risk ratio;

[1] One large multi-centre trial has examined the long term outcomes of caffeine therapy. This study was conducted in Australia, USA, Europe and Israel. The trial enrolled 2006 infants weighing 500g to 1250g who were treated with caffeine for a variety of indications. The data presented here are just for those who received caffeine for treatment of apnoea. There was no reason to downgrade the quality of evidence for risk of bias, or directness as the trials findings are applicable to similar neonates in all settings.
[2] It should be noted that this is a composite outcome. Overall the trial did not find evidence of a reduction in death, when examined alone.
[3] Downgraded for imprecision as the result is not statistically significant.
[4] Only one small study reported this outcome. Downgraded by 1 for risk of bias; as the method of allocation concealment was unclear, and downgraded by 2 for precision; as the 95% confidence interval is very wide and includes the possibility of a clinically important benefit or harm.
[5] No reason was found to downgrade for risk of bias, consistency, directness, or imprecision.
[6] Only two small studies have reported this outcome. Downgraded by 1 for risk of bias as the method of allocation concealment was unclear in both trials. The result is statistically significant.

Caffeine citrate compared to theophylline for treating apnoea

Patient or population: Preterm neonates
Settings: Low and middle income countries

Outcomes	Illustrative comparative risks* (95% CI)		Relative effect (95% CI)	No of Participants (studies)	Quality of the evidence (GRADE)	Comments
	Assumed risk	Corresponding risk				
	**Theophylline**	**Caffeine Citrate**				
**Death or major disability before 18 months**	-	-	-	(0 studies)	-	Not reported
**Cerebral palsy before 18 months**	-	-	-	(0 studies)	-	Not reported
**Chronic Lung Disease**	-	-	-	(0 studies)	-	Not reported
**Need for Mechanical Ventilation**	-	-	-	(0 studies)	-	Not reported
**Continuing apnoea at 1-3 days**	**125 per 1000**	**169 per 1000** (51 to 565)	**RR 1.35** (0.41 to 4.52)	45 (2 studies)	**low**[1]	
**Mean apnoea rate /100 mins at 1-3 days**	The mean apnoea rate at 1-3 days in the control groups ranged from **0.12 to 0.72 episodes/100 mins**	The mean apnoea rate at 1-3 days in the intervention groups was **0.11 episodes higher** (0 to 0.22 higher)		108 (5 studies)	**moderate**[2]	
**Continuing apnoea at 5-7 days**	**200 per 1000**	**300 per 1000** (64 to 1000)	**RR 1.5** (0.32 to 7.14)	20 (1 study)	**very low**[3]	
**Mean apnoea rate /100 mins at 5-7 days**	The mean apnoea rate at 5-7 days in the control groups ranged from **0.01 to 0.25 episodes/100 mins**	The mean apnoea rate at 5-7 days in the intervention groups was **0 episodes higher** (0.05 lower to 0.05 higher)		78 (4)	**moderate**[4]	
**Side effects**	**312 per 1000**	**53 per 1000** (13 to 225)	**RR 0.17** (0.04 to 0.72)	66 (3 studies)	**moderate**[5]	

*The basis for the **assumed risk** is the risk of the outcome in the groups treated with theophylline in the included trials.
The **corresponding risk** (and its 95% confidence interval) is based on the assumed risk in the comparison group and the **relative effect** of the intervention (and its 95% CI).
CI: Confidence interval; **RR:** Risk ratio;

[1] Only two very small studies reported this outcome. One study enrolled infants with a gestational age of < 31 weeks, and one enrolled pre-term infants with a mean gestational age of 30 weeks. Both trials adequately concealed allocation to be considered at low risk of bias. Neither trial found a statistically significant difference. Downgraded by 2 for imprecision as the 95% CI is very wide and includes the possibility of clinically important benefit or harm.
[2] The 5 studies reporting this outcome were conducted in preterm infants with a mean age below 32 gestational weeks. Allocation concealment was adequate in 2. Downgraded by 1 under precision as the 95% CI includes the possibility of no difference between treatments.
[3] Only one very small study measured this outcome. This trial enrolled pre-term infants with a mean gestation of 30 weeks. Downgraded by 1 for study limitations as the trial is severely underpowered to either achieve prognostic balance or to detect an effect. Also downgraded by 2 for imprecision as the 95% CI is very wide and includes the possibility of clinically important benefit or harm.
[4] The 4 studies reporting this outcome were conducted in preterm infants with a mean age below 32 gestational weeks. Allocation concealment was adequate in 2. Downgraded by 1 under precision due to the small size of the trials.
[3] The side effects were tachycardia or feed intolerance, and were consistently more common with theophylline across the three trials. Downgraded by 1 under precision due to the small size of the included trials.

Q2. What is the potential public health impact of applying the results to Ghana?

Neonatal deaths account for 60 percent of the deaths in infancy (GDHS 2008). Prematurity is noted to be the second major cause of infant mortality in Ghana (WHO 2007), and accounts for 25% of annual estimated neonatal deaths (WHO 2006).

Further estimation of the impact of the use of caffeine would require data on the incidence of neonatal apnoea in Ghana which was not available.

Q3. Is the current formulation suitable for Ghana?

Description of the formulation

**Route of administration:**	IV or Oral
**Additional requirements:**	**?**
**Storage:**	**?**
**Stability:**	**?**
**Transport:**	**?**

Is the introduction of this formulation feasible?

**Locally available manufacturers:**	None
**Ghana FDB Registration:**	None
**International manufacturers:**	None found
**Suggested level of prescribing:**	SD
**Educational requirements:**	
**System requirements:**	None
**Any other concerns:**	No

Will the introduction of this formulation be acceptable to all stakeholders?

**Toxicity:**	
**Appropriateness of formulation:**	
**Additional Stakeholders:**	Paediatricians/Neonatologists
**National Guidelines:**	There are currently no guidelines on the use of caffeine
**International Guidelines:**	

Q4. What are the resource implications?

What does this formulation cost?

	Formulation	Median	Minimum	Maximum
**IDPI Price Guide:**	None listed		-	-
**WHO Sources and Prices 2nd edition:**	None listed		-	-

Is it cost-effective?

We searched the Economic Evaluations database within the Cochrane library for evaluations of caffeine in the treatment of neonatal apnoea but none were found.

About the NHS Economic Evaluations Database within the Cochrane Library

As healthcare resources are finite, information about both costs and effects are essential to making evidence-based decisions about competing healthcare interventions. But information about cost-effectiveness can be difficult to identify, appraise and interpret.
The NHS Economic Evaluation Database (EED) assists decision-makers by systematically identifying economic evaluations from around the world, appraising their quality, and highlighting their relative strengths and weaknesses.
The NHS Economic Evaluations Database is produced by the Centre for Reviews and Dissemination (CRD) at the University of York, UK.

References

- Martin RJ, Abu-Shaweesh JM, Baird TM. Apnoea of prematurity. Paediatric Respiratory Review 2004;5:Suppl A:S377-S382
- WHO. *WHO Model List of Essential Medicines for Children* 3rd list. 2011. Available at: http://www.who.int/medicines/publications/essentialmedicines/en/index.html
- Ghana Ministry of Health. Ghana Essential Medicines List, Sixth edition. 2010. Ghana National Drugs Programme. Available at: http://ghndp.org
- Ghana National Health Insurance Scheme. Medicines List. 2011. Available at: http://www.nhis.gov.gh
- Ghana Ministry of Health. Ghana Standard Treatment Guidelines. Sixth edition. 2010. Ghana National Drugs Programme. Available at: http://ghndp.org
- Comer A. M, Perry C. M, Figgitt D. P, Caffeine citrate: a review of its use in apnoea of prematurity. Paediatric Drugs 2001; **3**(1):61–79.
- Henderson-Smart D. J, De Paoli A. G. Methylxanthine Treatment for Apnoea in Preterm Infants. Cochrane Database of Systematic Reviews 2010; Issue 12.
- Henderson-Smart D. J, Steer P. A. Caffeine versus Theophylline for Apneoa in Preterm Infants. Cochrane Database of Systematic Reviews 2010; Issue 1
- Barbara S, Roberts R. S, Davis P, Doyle L. W, Barrington K. J, Ohlsson A, et al. Caffeine Therapy for Apnoea of Prematurity. The New England Journal of Medicine 2006; 354 (20): 2112-21.
- Barbara S, Roberts R. S, Davis P, Doyle L. W, Barrington K. J, Ohlsson A, et al. Long-term Effects of Caffeine Therapy for Apnoea of Prematurity. The New England Journal of Medicine 2007; 357 (19): 1893-1902.
- Frye J. E. (ed.) International Drug Price Indicator Guide 2010 Management Sciences for Health & World Health Organisation 2011.
- Ghana Food & Drugs Board. List of Registered Products. Current Register March 2011.
- Ghana Statistical Service (GSS), Ghana Health Service (GHS), and ICF Macro. Ghana Demographic and Health Survey 2008. Accra, Ghana: GSS, GHS, and ICF Macro.
- NHIA. National Health Insurance Scheme Medicines List. March 2011.
- Dzadeyson E (ed.) WHO Study on maternal mortality and neonatal morbidity in Africa.
  Rural Integrated Relief Service-Ghana Journal; Publication date: 2007
  http://www.who.int/pmnch/topics/health_systems/rirs_ghana/en/ accessed 27th August, 2011
- WHO. Mortality Country Fact Sheet 2006. Annual estimated proportions of death by cause for neonates Ghana, 2000. http://www.who.int/whosis/mort/profiles/mort_afro_gha_ghana.pdf accessed 19th September 2011

Annex 1. Detailed search strategy and results

Set		Cochrane	PubMed
1		caffeine	caffeine[Title/Abstract]
2		apnoea	apnoea[Title/Abstract]
3		1 AND 2	1 AND 2
4			limit 3 to reviews or meta-analyses
**Search results**		**Cochrane**	**PubMed**
Hits		4	14
Included		2	2
Excluded		2	
Reason for exclusion	Topic not relevant to this summary		
	Not a systematic review		
	More complete reviews are available		
Additional reviews identified through reference lists			

Annex 2. AMSTAR assessment of the systematic review

Review reference: Henderson-Smart D J, De Paoli A G. Methylxanthine treatment for apnea in preterm infants. Cochrane Database of Systematic Reviews 2010, Issue 12.

**1. Was an 'a priori' design provided?**<br>The research question and inclusion criteria should be established before the conduct of the review.	☑ Yes<br>☐ No<br>☐ Can't answer<br>☐ Not applicable
**2. Was there duplicate study selection and data extraction?**<br>There should be at least two independent data extractors and a consensus procedure for disagreements should be in place.	☑ Yes<br>☐ No<br>☐ Can't answer<br>☐ Not applicable
**3. Was a comprehensive literature search performed?**<br>At least two electronic sources should be searched. The report must include years and databases used (e.g. Central, EMBASE, and MEDLINE). Key words and/or MESH terms must be stated and where feasible the search strategy should be provided. All searches should be supplemented by consulting current contents, reviews, textbooks, specialized registers, or experts in the particular field of study, and by reviewing the references in the studies found.	☑ Yes<br>☐ No<br>☐ Can't answer<br>☐ Not applicable
**4. Was the status of publication (i.e. grey literature) used as an inclusion criterion?**<br>The authors should state that they searched for reports regardless of their publication type. The authors should state whether or not they excluded any reports (from the systematic review), based on their publication status, language etc.	☐ Yes<br>☑ No<br>☐ Can't answer<br>☐ Not applicable
**5. Was a list of studies (included and excluded) provided?**<br>A list of included and excluded studies should be provided.	☑ Yes<br>☐ No<br>☐ Can't answer<br>☐ Not applicable
**6. Were the characteristics of the included studies provided?**<br>In an aggregated form such as a table, data from the original studies should be provided on the participants, interventions and outcomes. The ranges of characteristics in all the studies analyzed e.g. age, race, sex, relevant socioeconomic data, disease status, duration, severity, or other diseases should be reported.	☑ Yes<br>☐ No<br>☐ Can't answer<br>☐ Not applicable
**7. Was the scientific quality of the included studies assessed and documented?**<br>'A priori' methods of assessment should be provided (e.g., for effectiveness studies if the author(s) chose to include only randomized, double-blind, placebo controlled studies, or allocation concealment as inclusion criteria); for other types of studies alternative items will be relevant.	☑ Yes<br>☐ No<br>☐ Can't answer<br>☐ Not applicable
**8. Was the scientific quality of the included studies used appropriately in formulating conclusions?**<br>The results of the methodological rigor and scientific quality should be considered in the analysis and the conclusions of the review, and explicitly stated in formulating recommendations.	☑ Yes<br>☐ No<br>☐ Can't answer<br>☐ Not applicable
**9. Were the methods used to combine the findings of studies appropriate?**<br>For the pooled results, a test should be done to ensure the studies were combinable, to assess their homogeneity (i.e. Chi-squared test for homogeneity, I^2). If heterogeneity exists a random effects model should be used and/or the clinical appropriateness of combining should be taken into consideration (i.e. is it sensible to combine?).	☑ Yes<br>☐ No<br>☐ Can't answer<br>☐ Not applicable
**10. Was the likelihood of publication bias assessed?**<br>An assessment of publication bias should include a combination of graphical aids (e.g., funnel plot, other available tests) and/or statistical tests (e.g., Egger regression test).	☑ Yes<br>☐ No<br>☐ Can't answer<br>☐ Not applicable
**11. Was the conflict of interest stated?**<br>Potential sources of support should be clearly acknowledged in both the systematic review and the included studies.	☐ Yes<br>☐ No<br>☑ Can't answer<br>☐ Not applicable

For further information on the AMSTAR tool see: Shea B, Grimshaw J, Wells G, Boers M, Andersson N, Hamel C, et al. Development of AMSTAR: a measurement tool to assess the methodological quality of systematic reviews. BMC Medical Research Methodology. 2007; 7(1):10.

Review reference: Henderson-Smart DJ, Steer PA. Caffeine versus theophylline for apnoea in preterm infants. *Cochrane Database of Systematic Reviews* 2010, Issue 1. Art. No.: CD000273. DOI: 10.1002/14651858.CD000273.pub2.

**1. Was an 'a priori' design provided?**<br>The research question and inclusion criteria should be established before the conduct of the review.	☑ Yes<br>☐ No<br>☐ Can't answer<br>☐ Not applicable
**2. Was there duplicate study selection and data extraction?**<br>There should be at least two independent data extractors and a consensus procedure for disagreements should be in place.	☑ Yes<br>☐ No<br>☐ Can't answer<br>☐ Not applicable
**3. Was a comprehensive literature search performed?**<br>At least two electronic sources should be searched. The report must include years and databases used (e.g. Central, EMBASE, and MEDLINE). Key words and/or MESH terms must be stated and where feasible the search strategy should be provided. All searches should be supplemented by consulting current contents, reviews, textbooks, specialized registers, or experts in the particular field of study, and by reviewing the references in the studies found.	☑ Yes<br>☐ No<br>☐ Can't answer<br>☐ Not applicable
**4. Was the status of publication (i.e. grey literature) used as an inclusion criterion?**<br>The authors should state that they searched for reports regardless of their publication type. The authors should state whether or not they excluded any reports (from the systematic review), based on their publication status, language etc.	☐ Yes<br>☑ No<br>☐ Can't answer<br>☐ Not applicable
**5. Was a list of studies (included and excluded) provided?**<br>A list of included and excluded studies should be provided.	☑ Yes<br>☐ No<br>☐ Can't answer<br>☐ Not applicable
**6. Were the characteristics of the included studies provided?**<br>In an aggregated form such as a table, data from the original studies should be provided on the participants, interventions and outcomes. The ranges of characteristics in all the studies analyzed e.g. age, race, sex, relevant socioeconomic data, disease status, duration, severity, or other diseases should be reported.	☑ Yes<br>☐ No<br>☐ Can't answer<br>☐ Not applicable
**7. Was the scientific quality of the included studies assessed and documented?**<br>'A priori' methods of assessment should be provided (e.g., for effectiveness studies if the author(s) chose to include only randomized, double-blind, placebo controlled studies, or allocation concealment as inclusion criteria); for other types of studies alternative items will be relevant.	☑ Yes<br>☐ No<br>☐ Can't answer<br>☐ Not applicable
**8. Was the scientific quality of the included studies used appropriately in formulating conclusions?**<br>The results of the methodological rigor and scientific quality should be considered in the analysis and the conclusions of the review, and explicitly stated in formulating recommendations.	☑ Yes<br>☐ No<br>☐ Can't answer<br>☐ Not applicable
**9. Were the methods used to combine the findings of studies appropriate?**<br>For the pooled results, a test should be done to ensure the studies were combinable, to assess their homogeneity (i.e. Chi-squared test for homogeneity, I^2). If heterogeneity exists a random effects model should be used and/or the clinical appropriateness of combining should be taken into consideration (i.e. is it sensible to combine?).	☑ Yes<br>☐ No<br>☐ Can't answer<br>☐ Not applicable
**10. Was the likelihood of publication bias assessed?**<br>An assessment of publication bias should include a combination of graphical aids (e.g., funnel plot, other available tests) and/or statistical tests (e.g., Egger regression test).	☐ Yes<br>☑ No<br>☐ Can't answer<br>☐ Not applicable
**11. Was the conflict of interest stated?**<br>Potential sources of support should be clearly acknowledged in both the systematic review and the included studies.	☑ Yes<br>☐ No<br>☐ Can't answer<br>☐ Not applicable

For further information on the AMSTAR tool see: Shea B, Grimshaw J, Wells G, Boers M, Andersson N, Hamel C, et al. Development of AMSTAR: a measurement tool to assess the methodological quality of systematic reviews. BMC Medical Research Methodology. 2007; 7(1):10.

Annex 3. Assessment of the local applicability of the systematic review (SUPPORT tool 9)

Review reference: Henderson-Smart D J, De Paoli A G. Methylxanthine treatment for apnea in preterm infants. Cochrane Database of Systematic Reviews 2010, Issue 12.

1. Were the studies included in this systematic review conducted in settings similar to Ghana, or were the findings consistent across settings and time periods?

The studies included in this systematic review were conducted in developed countries such as United States, Europe, Canada, Australia and Israel. By this, the settings are different from Ghana; being a developing country. However, the findings can be considered to be applicable across settings as apnoea probably affects preterm infants in the same way worldwide.

2. Are there important differences in on-the-ground realities and constraints in Ghana that might substantially alter the feasibility and acceptability of this drug/formulation?

The trials were conducted in developed countries with good and well established health systems (adequate health personnel, modern equipments, good monitoring systems etc). On the contrary, neonatal intensive care in Ghana may be less readily available and affordable to the general population, especially in the rural areas. The introduction of caffeine citrate in treating apnoea in preterm neonates would require training and educating health personnel (doctors, nurses, medical assistants etc) on the appropriate application and monitoring processes.

3. Are there important differences in health system arrangements that may mean this drug/formulation could not work in the same way?

By the health system arrangement in Ghana, the lower level facilities should refer cases they cannot manage to the higher facilities. Premature infants require prompt intensive care to avoid complications and ensure their survival. Such lower facilities as the maternity homes and health centres are mostly in the rural areas with inadequate means of transport.

Again, intensive neonatal care units are not available at most lower facilities in the country.

4. Are there important differences in the baseline conditions that might yield different absolute effects even if the relative effectiveness was the same?

Generally, neonatal and infant mortality rates are higher in Ghana than the developed countries where the trials were conducted; which might affect the absolute effects obtained.

5. What insights can be drawn about options, implementation, and monitoring and evaluation?

The review of the trials indicates that caffeine treatment was beneficial to infants born at lower gestational ages but no analysis was done on the different gestational ages of the infants to know the impact; which needs to be further investigated.

For further information on the SUPPORT tool used for this assessment see: Lavis JN, Oxman AD, Souza NM, Lewin S, Gruen RL, Fretheim A. SUPPORT Tools for evidence-informed health Policymaking (STP) 9: Assessing the applicability of the findings of a systematic review. Health Res Policy Syst 2009, 7 Suppl 1:S9
